# Integrating Pharmacokinetics and Quantitative Systems Pharmacology Approaches in Generative Drug Design

**DOI:** 10.1021/acs.jcim.5c00107

**Published:** 2025-05-09

**Authors:** Helle W. van den Maagdenberg, Jikke de Mol van Otterloo, J. G. Coen van Hasselt, Piet H. van der Graaf, Gerard J. P. van Westen

**Affiliations:** † Leiden Academic Centre of Drug Research, Leiden University, 2333, Leiden, The Netherlands; ‡ Certara, CT2 7FG Canterbury, U.K.

## Abstract

Integrated understanding of pharmacokinetics (PK) and pharmacodynamics (PD) is a key aspect of successful drug discovery. Yet in generative computational drug design, the focus often lies on optimizing potency. Here we integrate PK property predictions in DrugEx, a generative drug design framework and we explore the generated compounds’ PD through simulations with a quantitative systems pharmacology (QSP) model. Quantitative structure–property relationship models were developed to predict molecule PK (clearance, volume of distribution and unbound fraction) and affinity for the Adenosine A_2A_R receptor (A_2A_R), a drug target in immuno-oncology. These models were used to score compounds in a reinforcement learning framework to generate molecules with a specific PK profile and high affinity for the A_2A_R. We predicted the expected tumor growth inhibition profiles using the QSP model for selected candidate molecules with varying PK and affinity profiles. We show that optimizing affinity to the A_2A_R, while minimizing or maximizing a PK property, shifts the type of molecular scaffolds that are generated. The difference in physicochemical properties of the compounds with different predicted PK parameters was found to correspond with the differences observed in the PK data set. We demonstrated the use of the QSP model by simulating the effect of a broad range of compound properties on the predicted tumor volume. In conclusion, our proposed integrated workflow incorporating affinity predictions with PKPD may provide a template for the next generation of advanced generative computational drug design.

## Graphical abstract

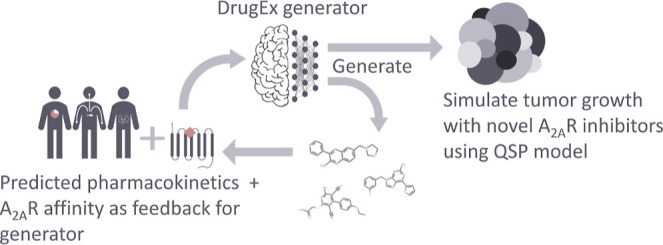

## Introduction

A lack of efficacy or safety is a major cause of drug failure.^1^ Drug efficacy is dependent on both pharmacokinetics (PK) and pharmacodynamics (PD). This makes the prediction of drug dose and PK a main concern during (model-based) drug development.^2,3^ Generative *de novo* drug design is used to efficiently explore the vast drug-like chemical space in early drug discovery.^4,5^ However, research in *de novo* drug design tends to focus on optimizing potency.^6–9^ Therefore, one of the key areas that needs to be addressed in generative artificial intelligence (AI) in drug discovery is the incorporation of PK and PD. To describe drug PK and PD, generally, PKPD and quantitative systems pharmacology (QSP) models can be used.^10^ A thorough mechanistic understanding of the biological system is required to understand the relationship between PK and PD. QSP models can describe the relationship between receptor activation and biomarkers for efficacy and toxicity. Consequently, the integration of PKPD and QSP modeling could play a role in bridging the gap between potency and efficacy in *de novo* drug design.

Several studies have explored the integration of PK or absorption, distribution, metabolism and elimination (ADME) in early drug discovery computational methods. For example, (*posthoc*) filtering for ADME properties has been applied in virtual screening and *de novo* drug design.^11,12^ Moreover, Horne et al.^13^ proposed simultaneous optimization of central nervous system (CNS) ADME properties, toxicity and potency through a generative machine learning method. A direct approach to the integration of PK into a generative AI framework was to generate novel compounds with increased CNS exposure through the use of physiologically based pharmacokinetic (PBPK) modeling and quantitative structure–property relationship (QSPR) models for the estimation of PK parameters as input to the generative model in a reinforcement learning framework.^14^ Yet, the sole focus here was optimizing PK, rather than the combination of PK and PD. Another tool, the commercial AI-driven drug design platform (AIDD)^15^ integrates ADME and PK properties directly into generative drug design. This tool allows users to integrate ADME and potency predictions into an evolutionary algorithm for *de novo* drug design. However, they do not link the predicted potency and PK properties of generated compounds to the PD and this tool is not open-source which limits usability by academics.

Illustrating the importance of combining PK and PD, Chen et al. developed a computational approach called model-based target pharmacology assessment.^3^ This method combines physiologically based pharmacokinetic (PBPK) and PD (specifically QSP) modeling with predictive models for ADME and potency to acquire a thorough understanding of the pharmacological system and drive decisions in the drug discovery pipeline.^3,16^ It emphasizes the complex relationship of the biological system with the PK and potency of compounds. In one of the described use cases the system is explored through simulations of the model-based system with a reaction-based virtual enumeration of compounds on a known scaffold,^16^ however, the authors did not explore the combination of generative AI and PK/QSP.

Here we aim to integrate PK into a generative drug design framework while considering the PD of the novel generated drugs. We simultaneously optimize target affinity and PK of small molecules in an AI-based *de novo* drug design framework. Subsequently, we explore the effect of the balanced optimization on predicted compound PD using simulations of a QSP model in a case study focused on the adenosine A_2A_ receptor (A_2A_R), a drug target in immuno-oncology. More specifically we will focus on optimizing clearance (CL), volume of distribution at steady state (VDSS) and the fraction unbound (FU), three fundamental PK properties.

First, we generated novel A_2A_R inhibitors using the multiobjective *de novo* drug design framework DrugEx^17^ with QSPR models for target affinity and for each PK property (CL, VDSS, FU). The optimization criteria here were to either maximize or minimize the respective PK property in addition to maximization of the affinity. While this does not directly reflect the optimization criteria during drug discovery/development, it allows us to analyze the effect of the optimization for certain PK properties in isolation, e.g. through comparison with high/low PK value compounds in the data set. Finally, we compared predicted tumor inhibition using an A_2A_R QSP^18^ model for molecules generated with only target affinity as optimization criteria versus molecules generated with PK and target affinity as criteria. Figure 1 shows a graphical representation of the proposed workflow.

**Figure 1:**
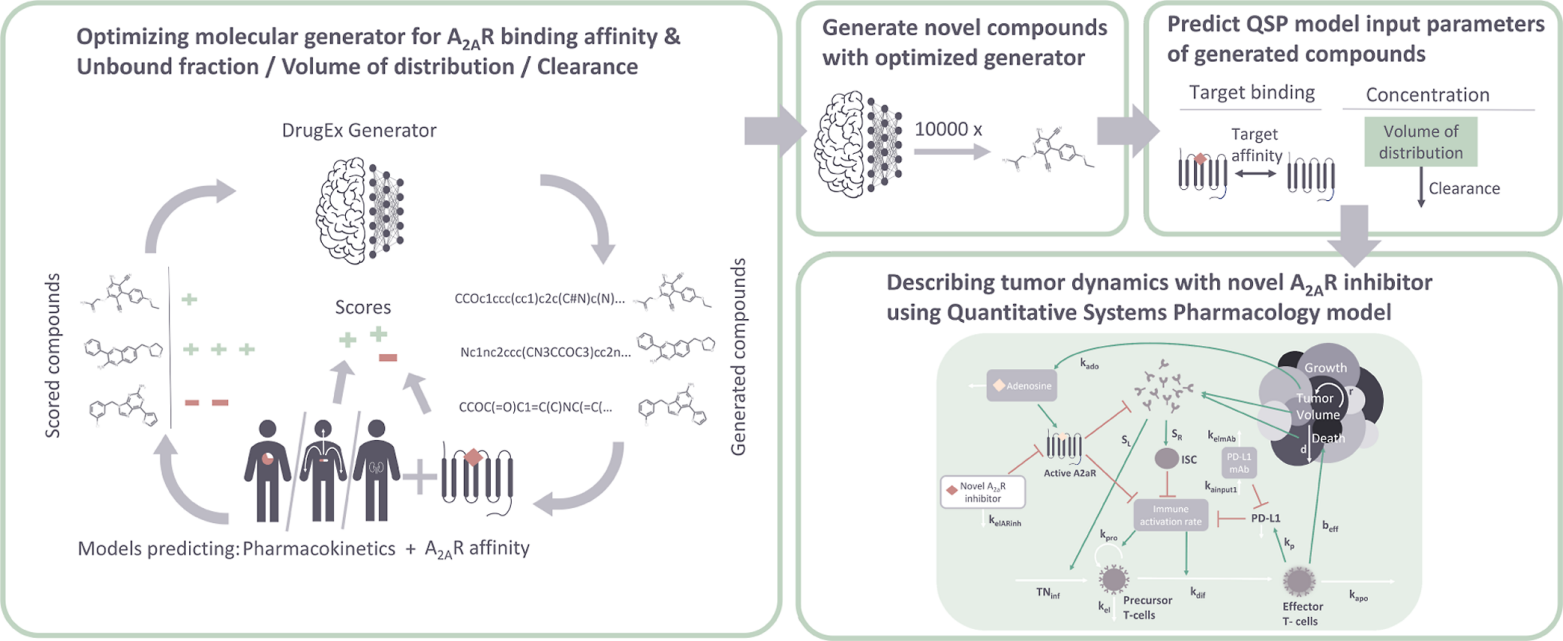
Overview of the workflow for *de novo* drug design described in this paper. First, a DrugEx molecular generator is trained to generate compounds with activity for the A_2A_R and a specified pharmacokinetic profile through reinforcement learning. It is trained to either maximize or minimize a specified pharmacokinetic property (CL, VDSS or FU). Then, the trained models are used to generate 10,000 compounds each. Finally, QSPR models are used to predict A_2A_R target affinity, VDSS and CL of the novel compounds, which form the input for a QSP model (adapted from ref 18). This model dynamically simulates the drug effect on the tumor microenvironment.

## Methods

### Data Collection

#### A_2A_R Data Set

Human bioactivity data for the A_2A_R (UniProt accession P29274) was extracted from Papyrus version 05.6,^19^ a large-scale curated bioactivity data set. The data was filtered to include only p*K*_i_ activity values and high-quality (as defined in Papyrus^19^) data points. Any molecules containing thiophene, amiloride (likely allosteric binding) or ribose (likely agonist) were removed. Molecules containing selenium were also removed for which not all descriptors could be calculated. Mean bioactivity values were used when multiple measurements were included in the data set. Figure S1 shows the agreement between multiple measurements. The preprocessed data set contained 3318 data points.

#### PK Data Set

PK data was extracted from a data set published by Lombardo et al.^20^ This data set contains a total of 1352 drugs with human PK parameters clearance in plasma (CL), steady-state volume of distribution (VDSS), and the fraction unbound in plasma (FU). Large molecules (molecular weight higher than 900 Da) and molecules containing metal atoms Pt (platinum) or Gd (gadolinium) were removed. CL and VDSS values were log-transformed, and FU values were square-root transformed to reduce the right-skewness of the data (see Figure S2). Stereochemistry was removed from the SMILES sequences for comparability with the A_2A_R data set. The data set was split into individual data sets for each property. The filtered data sets consisted of 1239 data points for CL, 1207 for VDSS, and 860 for FU. All preprocessed data can be found in Zenodo (https://zenodo.org/records/15082627).

### QSPR Model Training

QSPR models were developed with QSPRpred^21^ v3.0.2 (ChemProp^22^ models used v3.2.1). A grid search was performed over different data preprocessing options, models, and hyper-parameters for each of the four data sets. Data preprocessing steps always included SMILES standardization and salt stripping with the ChEMBL Structure Pipeline version 1.2.2.^23^ The RDKit^24^ (v2023.9.5) Morgan fingerprints (2048 bits, radius 3) and physicochemical 2D descriptors were explored as molecular representations. An independent test set (20%) was created with a random split. A standard scaler, fitted on the training set, was used to normalize the feature matrix. Features were filtered with either a low variance filter or the all-relevant feature selection method Boruta,^25,26^ combined with a high correlation filter. Scikit-learn K-nearest neighbor, random forest, and support vector machine models^27^ (v1.4.0) and ChemProp^22^ (v1.6.1) models with different hyper-parameters were examined. See Table 1, for an overview of the hyper-parameter grid. Different combinations of preprocessing steps and models were compared using 5-fold cross-validation. The best combination was selected based on the highest mean coefficient of determination (*R* ^2^) over the cross-validation folds. For efficiency, model hyper-parameter grid search was stopped if a search lasted more than 8 h, which was the case for some combinations of data sets (with a large number of descriptors and samples) and support vector machine models (see Table S2). The model performance was assessed on the independent test set. Feature importance was evaluated using permutation importance as implemented in scikit-learn^27^ (v1.4.0) with 30 repeats.

**Table 1:** Grid Search Parameter Grid Including Different Data Pre-Processing Steps and Model Types

step	hyperparameters	values
Features		
	descriptors sets	RDKit, MorganFP, RDKit & MorganFP
Filters		
high correlation	threshold	0.9, 0.95, 0.99
low variance	threshold	0.01, 0.05, 0.1
boruta filter	percentage	50, 80, 100
Models		
random forest	n_estimators	100, 300, 500, 1000
	max_depth	5, 10, 20
support vector machine	C	0.1, 1, 10
	kernel	linear, rbf
partial least-squares	n_components	5, 7, 10, 20, 50
chemprop	depth	3, 5
	hidden_size	128, 256, 512
	ffn_num_layers	1, 2, 3
	dropout	0.0, 0.1, 0.2

### Applicability Domain

The best data preprocessing/model combination was used on different bootstraps of the whole data set to assess the applicability domain of the trained models and further investigate the model performance. For the bootstrapping, the data set was split using a random split or cluster split (20%) for 50 replicas each. The applicability domain of each replicate was estimated using the TOPKAT Optimum Predictor Space (OPS)^28^ as implemented in MLChemAD.^29^ Briefly, here the OPS was defined as the orthogonal projections via eigenvalue decomposition of the min–max normalized data set. A new observation is considered outside of the applicability domain if the Mahalanobis distance of the observation in the OPS is larger than 1.5 times the number of dimensions divided by the number of observations. Each training set was used to fit and subdivide the test set into inliers and outliers. Every replicate was assessed on the *R* ^2^, and root mean squared error (RMSE) for the total test set.

### DrugEx Training

A DrugEx recurrent-neural net (RNN) model^17,30^ pretrained on Papyrus version 05.5 ^31^ was used as a baseline model. DrugEx (v3.4.7) was used for model training. Through transfer learning, this model was finetuned to generate molecules close to the known data for the respective data sets. Seven data sets were created for finetuning, one for each objective and one for each combination of the A_2A_R and a PK property. For each objective (A_2A_R, Cl, VDSS, FU), finetuning was applied to all molecules applicable according to the respective OPS. For combinations of A_2A_R and a PK objective, the model was finetuned on all data from the A_2A_R data set that was applicable according to the PK and A_2A_R OPS. Each model was finetuned for a maximum of 500 epochs with early stopping based on validation loss of a randomly selected 10% subset of the data. For the reinforcement learning, scores for each objective were normalized to fall between 0 and 1 using a clipped scoring strategy, where scores below and above a certain threshold are set to 0 and 1 respectively and the values are scaled linearly between the thresholds. See Table S1 for details. Thresholds were set to the 10th and 90th percentiles of the property values in the respective data set. Thirteen scenarios for optimization were configured: maximizing A_2A_R, maximizing/minimizing CL, VDSS or FU, maximizing A_2A_R, and maximizing/minimizing CL, VDSS or FU. For each of the scenarios, reinforcement learning was run for 2000 epochs, with early stopping with a patience of 300 epochs and a minimum of 200 epochs using a batch size of 512 for 3 replicas. Here, the finetuned model was used as the agent (updated during training). A grid search was performed to find optimal values for epsilon (rate of mutation) and which network to use as the mutation network (fixed during training).^17^ The results of the grid search can be found in Table S3. The fine-tuned model was selected as the mutation network and a value of 0.1 was selected for epsilon. The stopping criteria was the improvement in the arithmetic mean of the objectives scores. To determine the reward in the reinforcement learning framework ranking by Pareto front and subranking with Tanimoto distance was used.^17,30^ After optimization for each scenario and replicate, 10,000 molecules were generated, which were evaluated on validity, uniqueness, novelty, and chemical diversity.

### Quantitative Systems Pharmacology Model

To simulate the *in vivo* effect of the novel structures, a previously published QSP model^18^ was used. This model captures tumor-cell dynamics in mice syngeneic models with the A_2A_R inhibitor, AZD4635, alone and in combination with an anti-PD-L1 specific antibody. Here, we replaced the AZD4635 PK and A_2A_R binding with the predicted PK of our generated molecules to evaluate the effect of different reinforcement scenarios. As the QSP model was developed with mice models, the predicted human Cl and VDSS parameters were scaled using allometric scaling according to the equation given below, assuming an allometric exponent of 0.65 for CL^32^ and 0.95 for VDSS.^33^ Using eq 1 to scale the parameters.

1 $$P_{mice}=P_{human}/{\left({BW}_{human}/{BW}_{mice}\right)}^{\alpha }$$

Here, *P* represents the scaled parameter (CL/VDSS), α is the allometric exponent, and BW is body weight (0.025 kg for mice/70 kg humans).

The model code provided by Voronova et al.^18^ was reproduced with RxODE2 (v2.1.2).^34^ The 2-compartment oral absorption model for AZD4635 was replaced by the following 1-compartment model with intravenous administration as given in equations. In the model by Voronova et al.,^18^ the effect was based on the total concentration of AZD4635, therefore, the predicted unbound fraction was not used.

2a $$\frac{d}{dt}\left(Ac2\right)=-ke{l_{ARinh}}^{*}Ac2$$

2b $$Cc2=\frac{Ac2}{MW\times Vc}$$

Here, Ac2 is the amount and Cc2 the concentration of the A_2A_R inhibitor in the central compartment. MW is the molecular weight, kel_ARinh_ the predicted elimination rate constant, and *V*c the predicted volume of distribution in the central compartment of the *de novo* generated A_2A_R inhibitors.

Voronova et al. determined covariates for the four different studies investigated; here we selected to use the covariates from the MCA205-2 syngeneic mice model study (sL_cov_ = 0, TVin_cov_ = 0.69, Vado_cov_ = −3, sR_cov_ = 0.5308). Where sL is the T-cell’s ability to infiltrate tumor tissue under systemic antigen exposure, TVin the initial tumor volume, Vado is the average adenosine level in the tumor and sR sensitivity of cellular immunosuppression. We selected to use the MCA205-2 syngeneic mice model study as this model showed higher sensitivity to A_2A_R inhibitor^18^ due to the lower average adenosine levels in the tumor. The complete set of model equations (eq S1), parameters (Table S5) and variable definitions (Table S4) may be found in the Supporting Information.

For each set of generated compounds, the tumor volume over time was simulated with the same dosing scheme used in the simulations in Voronova et al.^18^ of anti-PD-L1 mAb at 5 mg/kg twice weekly and AZD4635 50 mg/kg twice daily. The system was simulated for 30 days (starting from tumor inoculation), with drug dosing between 7 and 22 days.

## Results

### PK and Affinity Prediction Accuracy

To achieve integration of drug PK in *de novo* drug generation, we utilized public data sets to train QSPR models for the prediction of drug PK and potency. After data collection and preprocessing, a grid search was performed on different features, feature filters, model algorithms, and hyperparameters (Table S2). For all models, the difference in performance on the 20% holdout set and cross-validation test sets is small (<0.15 on the *R* ^2^) (Figure 2). This shows that there is no large overfitting effect present due to feature and hyperparameter selection. The importance of individual features was analyzed using permutation importance (Figure S3).

**Figure 2:**
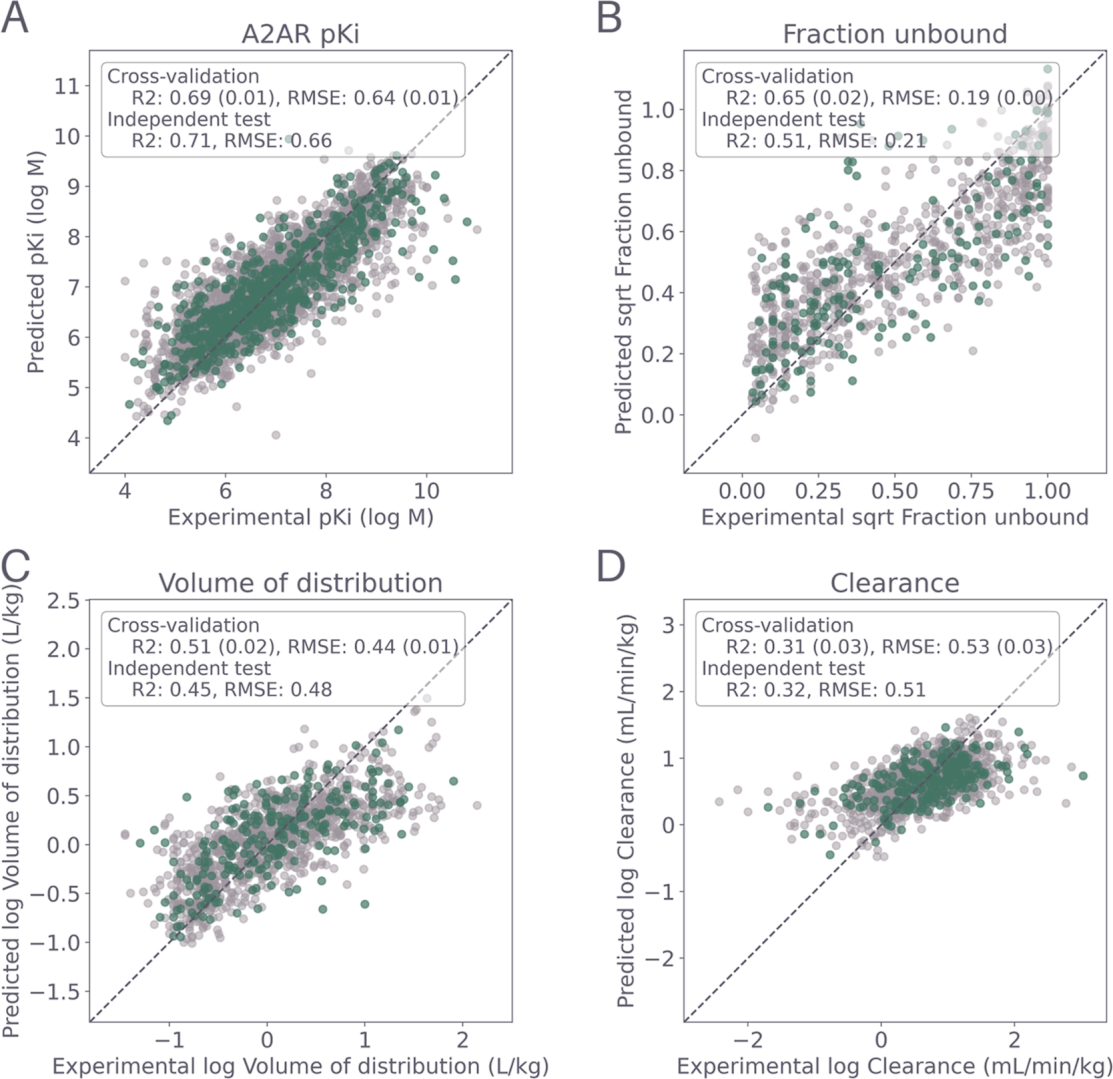
Scatter plots of observed (*x*-axis) versus predicted (*y*-axis) values showing the performance of best QSPR models for (A) A_2A_R binding affinity, (B) CL, (C) FU, and (D) VDSS on the 5-fold cross-validation validation sets (light gray) and the independent test set (green). Performance metrics R^2^ and RMSE are noted in the corresponding figures. Mean *R* ^2^ and RMSE values are used for the cross-validation test sets.

While the random hold-out test set provides an impression of the model performance, the results depend on the initial random seed. A bootstrap analysis was performed to analyze the model performance more extensively, where the data set was split randomly 50 times (80%/20%). The mean bootstrapping *R* ^2^ for A_2A_R pKi is 0.70 (range 0.66–0.74) and the RMSE is 0.63 (range 0.58–0.68) (Figure 3). The bootstrapping results affirm that the model for CL (*R* ^2^ 0.34 (0.26–0.43)) performs worse than the models for the other PK properties FU (*R* ^2^ 0.64 (range 0.57–0.72)) and VDSS (0.50 (range 0.42–0.57)).

**Figure 3:**
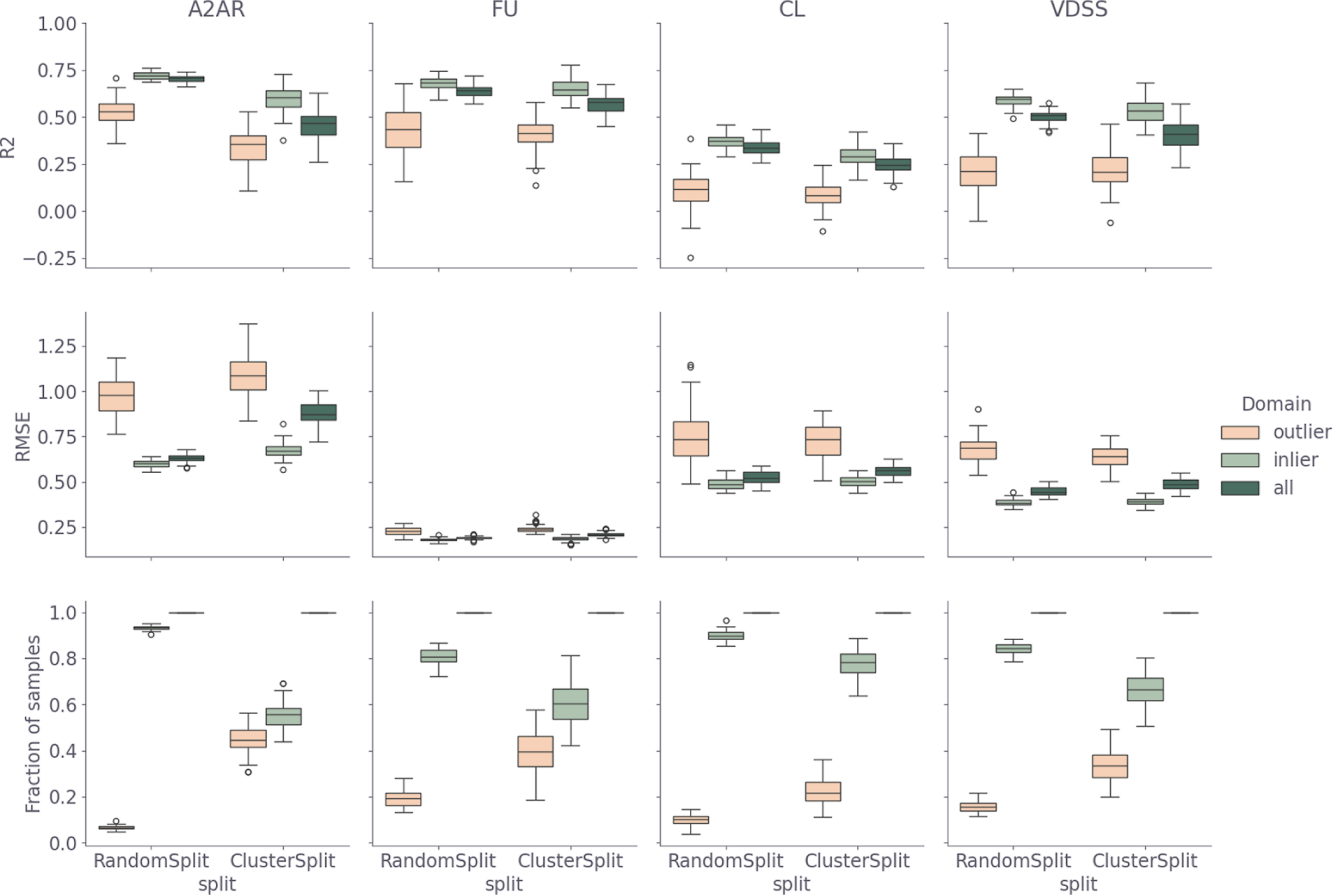
Bootstrapping performance of the QSPR models for 50 random splits and 50 cluster splits for the outliers (peach), the inliers (light green), and the complete test set (dark green), respectively. Each row shows a different metric, *R* ^2^ and RMSE (calculated on transformed target properties) and the fraction of samples in the test set. The columns show the performance of the different target properties: A_2A_R binding affinity, FU, VDSS and CL.

To evaluate the models’ ability to make prospective predictions, the same procedure was repeated with 50x a balanced cluster split.^35^ As expected, a drop in the performance (lower *R* ^2^ and higher RMSE) occurs on average compared to the random split test set (Figure 3) for each target property. This drop is most pronounced for the A_2A_R pKi (mean R^2^ from 0.70 to 0.46). This may be due to the smaller diversity in the A_2A_R receptor set, compared to the PK set. Another possible cause is the higher initial performance and therefore more potential to decrease. Hence, there is less difference between the random split and the cluster split for the PK data set than the A_2A_R data set.

The data generated for the bootstrapping analysis with the two different random splits was also used to analyze the ability of the applicability domain to discriminate inliers from outliers. The OPS was determined on each training set and subsequently used to split the test set into inliers and outliers. The performance of the models was calculated separately for the inliers (light green) and the outliers (peach) as shown in Figure 3. Here, we observe that according to the OPS applicability domain, there is a higher fraction of outliers in the cluster split test sets than for the random split. This corresponds well with the stricter nature of the cluster split which increases the chemical distance between the train and test set. In general, the performance of the model is worse (lower *R* ^2^ and higher RMSE) on the outliers than on the inliers, for both the cluster split and random split test sets. Furthermore, a reduction in the difference in performance between the random split and cluster split test sets can be observed for the inliers compared to the complete test set performance. Therefore, we concluded that the OPS applicability domain is sufficient to distinguish outliers during reinforcement learning.

### *De Novo* Generation with Optimization for PK and PD

A DrugEx pretrained RNN generator was fine-tuned on different data sets of applicable molecules for the different optimization tasks (Figure S4). After fine-tuning, the models were optimized for the respective single (maximize/minimize A_2A_R/FU/VDSS/CL) or combined tasks (maximize A_2A_R + maximize/minimize FU/VDSS/CL) (Figure S5). Subsequently, each fine-tuned and reinforced model was used to generate 10,000 compounds. Validity, uniqueness, novelty (presence in the data set), applicability, synthetic accessibility, and chemical similarity of the generated compounds were determined and compared (Table 2). For the fine-tuned generators a slight drop in the fraction of valid (i.e., parsable by RDKit) molecules (0.75–0.87) compared to the pretrained generator (0.96) was found. This corresponds with the observed initial drop and gradual recovery of validity during fine-tuning (Figure S4). However, the fraction of valid compounds of the pretrained model is restored after reinforcement learning (0.91–0.99). This suggests increasing the patience in fine-tuning in future research may be beneficial for the validity of generated molecules. The uniqueness also decreases after fine-tuning, which reflects the smaller area of chemical space the model covers.

**Table 2:** Statistics of the Trained DrugEx Generators Reinforced with Different Objectives^a^

scenario	valid	valid & unique	valid & unique & AP	valid & unique & AP & novel	mean SA score	mean intra-data set minimum Tanimoto distance	mean inter-data set minimum Tanimoto distance
pretrained	0.96	0.96	0.96	0.95	2.79	0.66	∼
fine-tuned on A2AR	0.86	0.62	0.38	0.19	2.52	0.51	0.47
max A2AR	0.99 (0.00)	0.61 (0.01)	0.56 (0.00)	0.54 (0.00)	2.79 (0.02)	0.25 (0.00)	0.36 (0.00)
fine-tuned on FU	0.75	0.61	0.51	0.46	2.99	0.69	0.75
min FU	0.91 (0.01)	0.90 (0.01)	0.90 (0.01)	0.90 (0.01)	2.73 (0.08)	0.67 (0.02)	0.79 (0.01)
max FU	0.95 (0.01)	0.92 (0.02)	0.87 (0.02)	0.87 (0.02)	4.02 (0.12)	0.67 (0.02)	0.82 (0.01)
fine-tuned on VDSS	0.76	0.67	0.58	0.52	3.07	0.69	0.74
min VDSS	0.95 (0.01)	0.83 (0.03)	0.83 (0.03)	0.83 (0.03)	3.13 (0.06)	0.57 (0.01)	0.75 (0.00)
max VDSS	0.97 (0.00)	0.94 (0.00)	0.88 (0.00)	0.88 (0.0)	3.09 (0.03)	0.59 (0.01)	0.77 (0.00)
fine-tuned on CL	0.74	0.65	0.60	0.54	3.12	0.70	0.74
min CL	0.97 (0.00)	0.87 (0.05)	0.87 (0.05)	0.87 (0.05)	2.53 (0.07)	0.47 (0.02)	0.70 (0.01)
max CL	0.97 (0.00)	0.94 (0.00)	0.93 (0.01)	0.93 (0.01)	2.78 (0.03)	0.54 (0.00)	0.75 (0.00)
fine-tuned on A2AR + FU	0.87	0.62	0.36	0.20	2.44	0.52	0.50
max A2AR + min FU	0.99 (0.00)	0.67 (0.02)	0.59 (0.02)	0.58 (0.02)	2.59 (0.02)	0.25 (0.00)	0.41 (0.00)
max A2AR + max FU	0.98 (0.00)	0.64 (0.01)	0.37 (0.01)	0.37 (0.01)	2.91 (0.04)	0.35 (0.01)	0.57 (0.01)
fine-tuned on A2AR + VDSS	0.86	0.61	0.36	0.19	2.47	0.53	0.50
max A2AR + min VDSS	0.98 (0.00)	0.69 (0.01)	0.37 (0.01)	0.36 (0.01)	2.47 (0.04)	0.31 (0.01)	0.50 (0.00)
max A2AR + max VDSS	0.98 (0.00)	0.73 (0.01)	0.38 (0.02)	0.38 (0.02)	2.65 (0.02)	0.26 (0.01)	0.51 (0.01)
fine-tuned on A2AR + CL	0.85	0.62	0.37	0.20	2.48	0.52	0.49
max A2AR + min CL	0.99 (0.00)	0.69 (0.02)	0.37 (0.02)	0.37 (0.02)	2.55 (0.02)	0.29 (0.0)	0.49 (0.01)
max A2AR + max CL	0.99 (0.00)	0.62 (0.02)	0.34 (0.01)	0.33 (0.01)	2.62 (0.02)	0.30 (0.01)	0.53 (0.00)

a For each optimization scenario the fraction of valid molecules, the fraction of unique molecules, the fraction of applicable molecules (according to respective QSPR OPS applicability domains), the fraction of novel molecules (not contained in any of the training sets), the average synthetic accessibility score, mean internal (i.e., the mean distance of each molecule to its closest neighbor in the data set) and external distance is given. For the reinforced models the standard error of the mean of the three replicates is stated between brackets.

After reinforcement learning, the uniqueness decreases further for all optimization scenarios involving the A_2A_R data set. This data set is characterized by lower chemical diversity than the PK data set, which also limits the applicability domain of the QSPR model to a smaller part of the chemical space. The applicability domain thus confines the ability of DrugEx to explore new regions. Contrary to the valid and unique molecules, a higher fraction of novel (i.e., not presented in the fine-tuning data set) molecules are generated by the reinforced generators than by the fine-tuned generators. This shows that the reinforced generator is not replicating the data set molecules even though it generates quite similar molecules. The reduction in diversity is also confirmed by the mean minimum internal Tanimoto distance (i.e., the mean distance of each molecule to its closest neighbor in the data set), which is strongly reduced in reinforced (0.25–0.35) generators for maximizing the A_2A_R pKi compared to the pretrained model (0.66). For the mean minimum distances to the respective fine-tuning data set, generally no large increase or decrease in distance is observed after reinforcement learning, which suggest exploitation around the active compounds rather than exploration to completely new chemical space. This is expected, due to the applicability domain as optimization criteria. As the difference between replicates are small (SEM <0.1 for fraction of valid and unique & applicable and novel), only the first replicate was used for the following analyses of the results.

To evaluate the success of the optimization, the predicted properties’ distributions were compared (Figure 4). After optimization for A_2A_R affinity, a strong shift to higher predicted affinity (pKi) is achieved in the maximized model (μ 8.76, σ 0.51) compared to the fine-tuned model (μ 6.62, σ 0.86) (Figure 4A). A similar, although less pronounced shift is observed for the optimization of affinity in combination with the maximization/minimization of FU (Figure 4A) (μ 7.49, σ 0.61; μ 8.52, σ 0.46), VDSS (Figure 4B) (μ 7.61, σ 0.73; μ 7.30, σ 0.79), and CL (Figure 4C) (μ 7.65, σ 0.68; μ 7.49, σ 0.73). These results reflect the trade-off between the A_2A_R target affinity and the other objectives. Results for the individual PK optimization criteria can be found in the Supporting Information (Figure S6).

**Figure 4:**
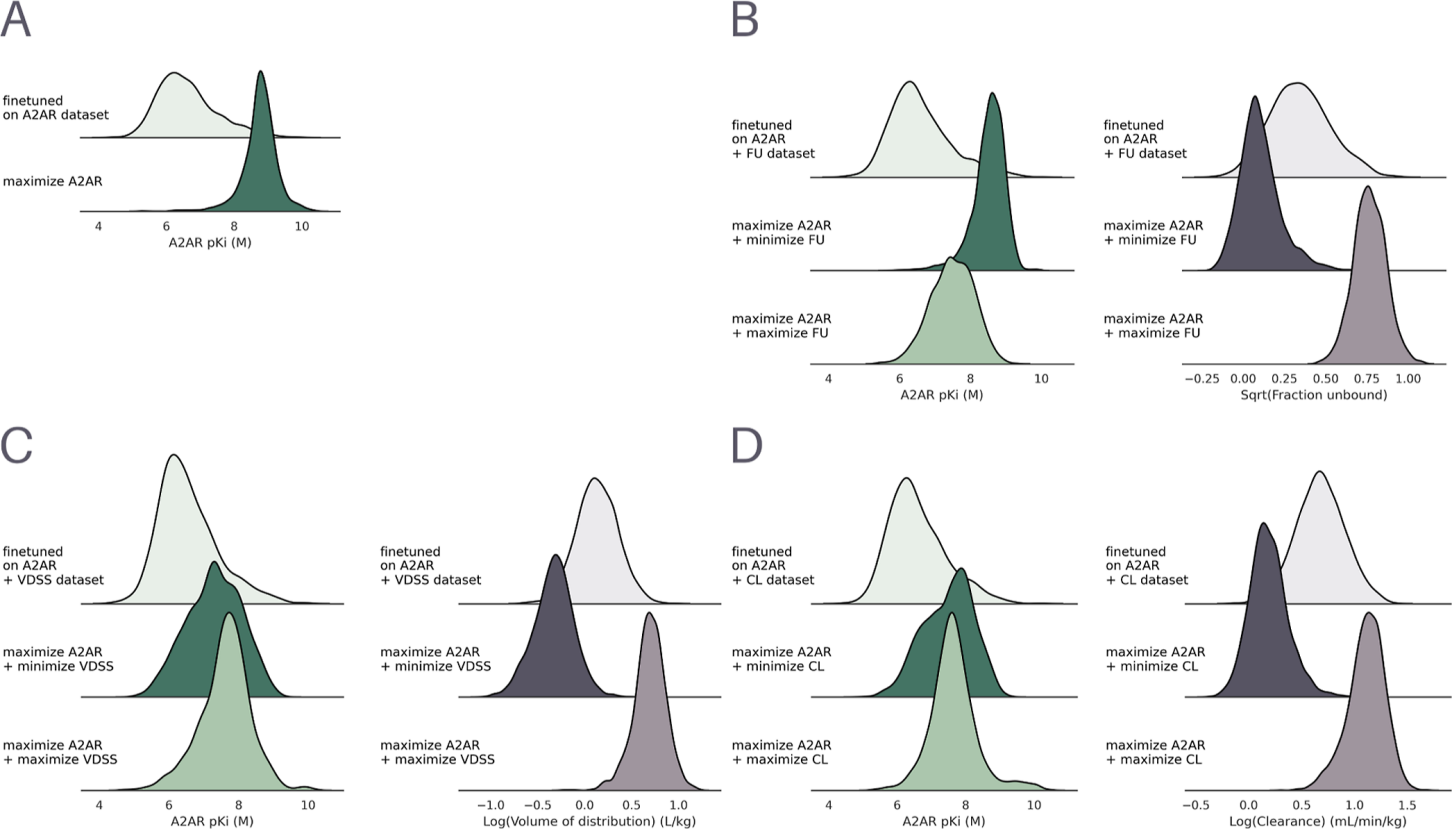
Distribution of scores of all valid and unique molecules from a set of 10,000 generated molecules for different DrugEx optimization scenarios. The panels show optimization for (A) maximization of A_2A_R pKi, (B) A_2A_R pKi + minimization/maximization of FU, (C) A_2A_R pKi + maximization/minimization of VDSS and (D) A_2A_R pKi + maximization/minimization of CL. The left column in each panel shows the A_2A_R pKi density and the right column shows the respective PK property density.

The plausibility of the generated molecules was assessed through visual inspection of a UMAP^36^ of the data set and the generated molecules combined 5. The fine-tuned generators match the distribution of the data set neatly (second row Figure 5). The distribution of the molecules generated by reinforced generators matches the areas with the respective high/low property values in the data set. Notably, the generated molecules cover different areas of the data set depending on the optimization objectives. UMAPs for the single PK objective generators are available in the Supporting Information (Figure S7).

**Figure 5:**
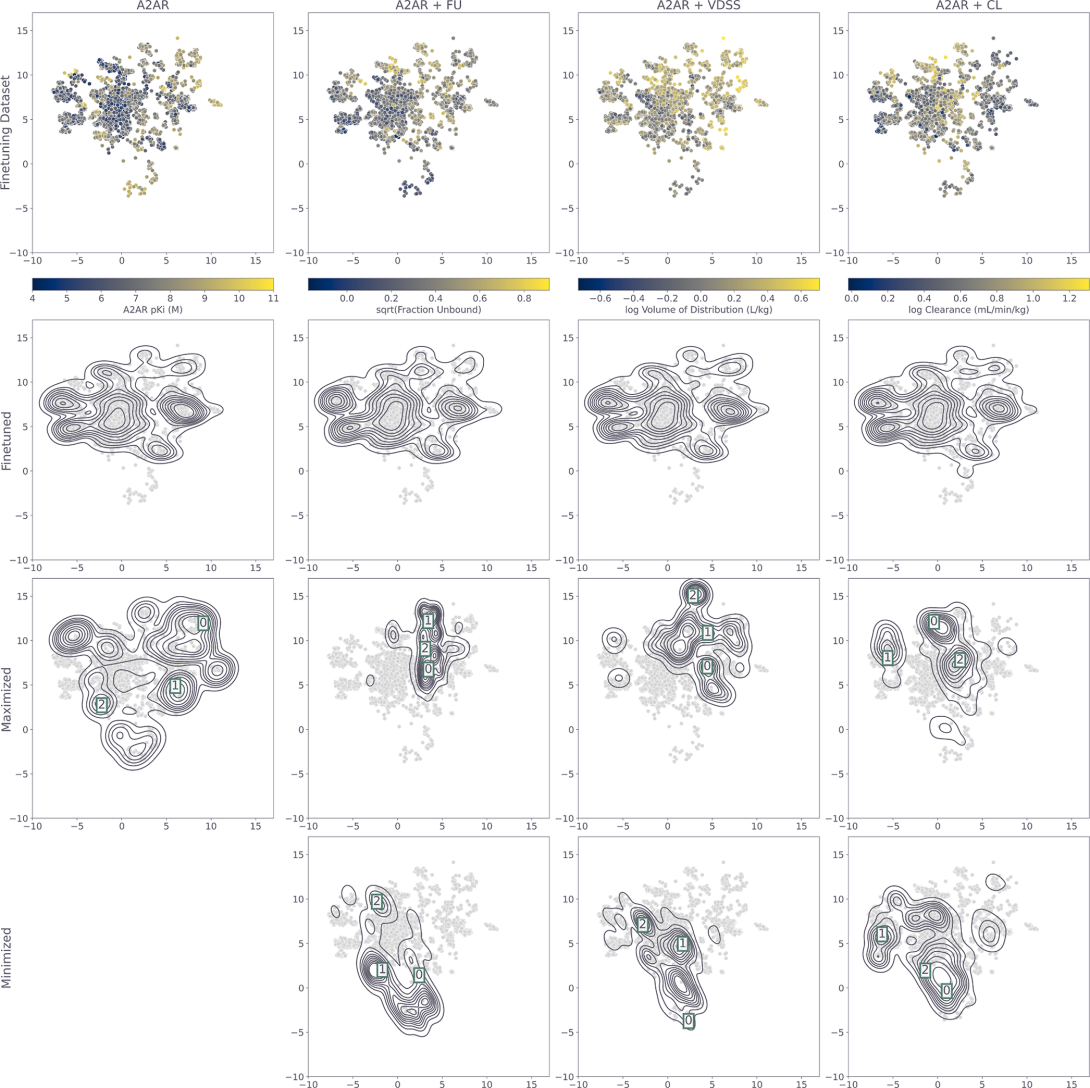
Umap representation of the A_2A_R data set overlapping with the density of the generated molecules from the trained DrugEx generators with different objectives. The columns represent the different combinations of properties. The first row shows the data set colored by the different properties from left to right: A_2A_R pKi (data set), sqrt FU, log VDSS, log CL (predicted); containing only the molecules applicable for that combination of objectives. The second row shows the density of all valid and unique molecules generated by the fine-tuned models. The third and fourth rows show the density of the unique and valid generated molecules for maximization of the A_2A_R pKi and maximization or minimization of a PK property, respectively. Square frames with numbers indicate the location of the molecules highlighted in Figure 6.

Images of the 2D generated molecule structures (Figure 6), selected through diversity clustering, show that most generated structures are similar to high-affinity molecules from the A_2A_R data set. Depending on the generation scenario, distinct types of scaffolds are generated. For example, in the maximized VDSS scenario, triazolo-pyrimidine and purine derivatives are more common than in the minimized VDSS scenario. Here, we did not use selectivity for the A_2A_R as optimization criteria, thus generated compounds may be similar to compounds with activity for other adenosine receptor subtypes. For example, compound 1 from the maximize A_2A_R + minimize CL scenario, where the data set corresponding data set compound is also active for the A_2B_R (pKi 6.7).^37^

**Figure 6:**
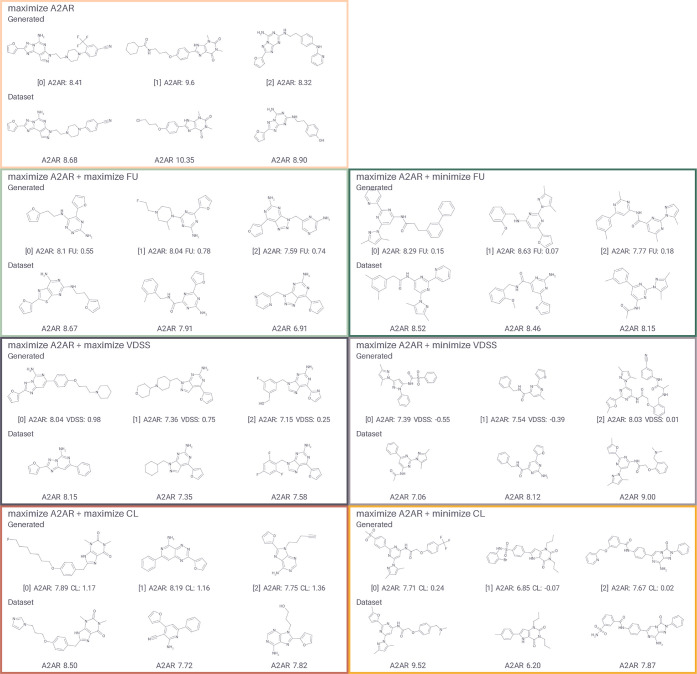
Example generated molecules and most similar data set compounds for different DrugEx optimization scenarios. Each box contains the centroids of the five largest clusters from leader picker cluster analysis (Tanimoto similarity threshold 0.8, Morgan fingerprints with radius 3, bits 2048) on the set of generated molecules. The first row shows the centroids, and the second row shows the most similar data set compounds by Tanimoto distance (Morgan fingerprints with radius 3, bits 2048.). Below each generated compound the predicted value for each relevant property is shown; below each data set compound the experimental mean A_2A_R pKi is shown.

Patterns in physicochemical properties of the generated compounds generally match what was observed in the score distribution and UMAPs, namely different distributions of physicochemical properties for the generated molecules in different scenarios (Figure 7). Furthermore, the scenario with maximized FU compared to only maximized affinity clearly shows a trend toward lower lipophilicity, lower molecular weight and higher fraction of sp^3^ hybridized carbons. This trend is reflected in the PK data set as well (Figure S8) in the difference between low and high FU compounds. This further confirms a trade-off between affinity for A_2A_R and FU. Moreover, for the maximization of the CL scenario, a decrease in molecular weight and increase in the fraction of sp^3^ hybridized carbons is noted, compared to the minimization scenario. This difference is also present in the low versus high CL compounds in the PK data set (Figure S8). For the maximization of VDSS scenario a lower polar surface area and higher fraction of sp^3^ hybridized carbons is found compared to the maximization; and again a similar difference is found in PK data set (Figures 7 and S8).

**Figure 7:**
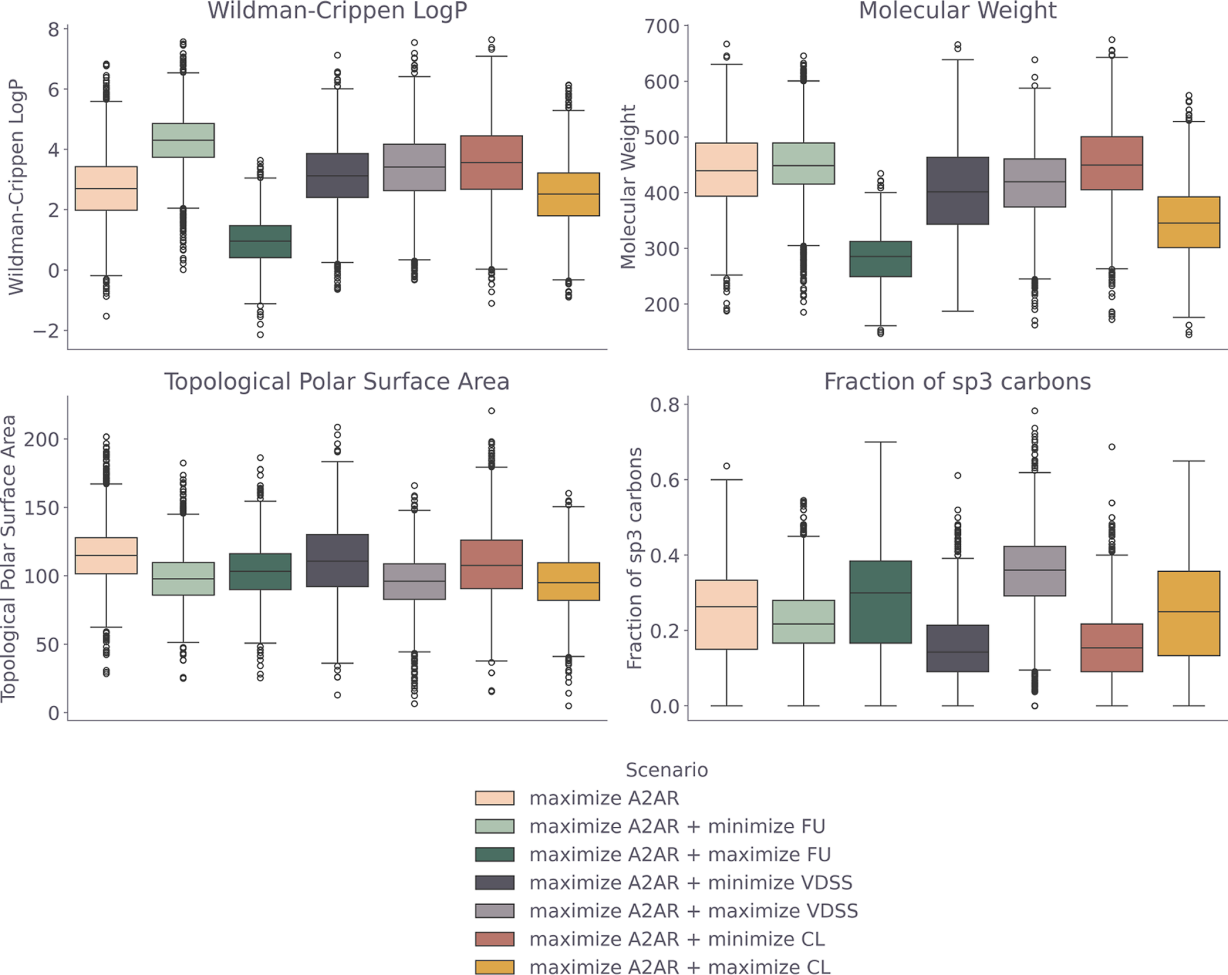
Box plots of the physicochemical properties (Wildman-Crippen LogP, Molecular Weight, Topological Polar Surface Area, Fraction of sp^3^-hybridized carbons) of all valid and unique molecules from a set of 10,000 generated molecules for different DrugEx optimization scenarios.

### QSP Model Simulations of *De Novo* Generated Compounds

Drug PD is not only dependent on potency, but on the interplay between the potency, PK and the biological system. Therefore, we use a QSP model to simulate the predicted PD of the generated compounds. To evaluate the influence of the different optimization scenarios on the eventual tumor inhibition effect, the properties of the generated compounds were used as input to a QSP model adapted from Voronova et al.^18^ This mice mechanistic model (Figure 8A) describes the influence of adenosine occupancy of the A_2A_R on the immune system, represented by the immune activation rate and systemic antigen. The immune activation rate stimulates the production of and differentiation of precursor T-cells, which in turn can increase tumor cell death. The systemic antigen has a dual role where it increases the influx of precursor T-cells, but immunosuppressive cells as well. The model also includes the effect of a PD-L1 mAb, which has a synergistic effect with inhibition of the A_2A_R.^18^ As the effect in this model is based on the total concentration of the inhibitor and not the unbound concentration, the scenarios with minimization/maximization of FU are not considered here. For the sets of generated compounds, the A_2A_R affinity, VDSS and CL were predicted and used for QSP model simulations. From simulations of the tumor volume over 30 days with dosing of the generated A_2A_R inhibitors and a PD-L1 mAb, the 90% prediction interval and mean prediction of the typical individual were plotted for each of the scenarios (Figure 8C). These show that decreasing exposure by maximization of CL and minimization of VDSS, decreases the effectiveness of the drug. However, the increased exposure by minimization of CL and maximization of VDSS, does not compensate for the decrease in potency by only maximizing the A_2A_R affinity. Therefore, these simulations can help in identifying the most promising drug candidates. Simulations with four selected compounds with extreme (highest or lowest elimination rate compound in the respective bottom and top 10% quantile of potency) values show that both favorable PK as well as good potency are necessary for achieving sufficient tumor inhibition (Figure 8B). The reason that no increased tumor inhibition is observed for the simulations with maximization of VDSS and minimization of CL is that optimization for either property also increases/decreases the other.

**Figure 8:**
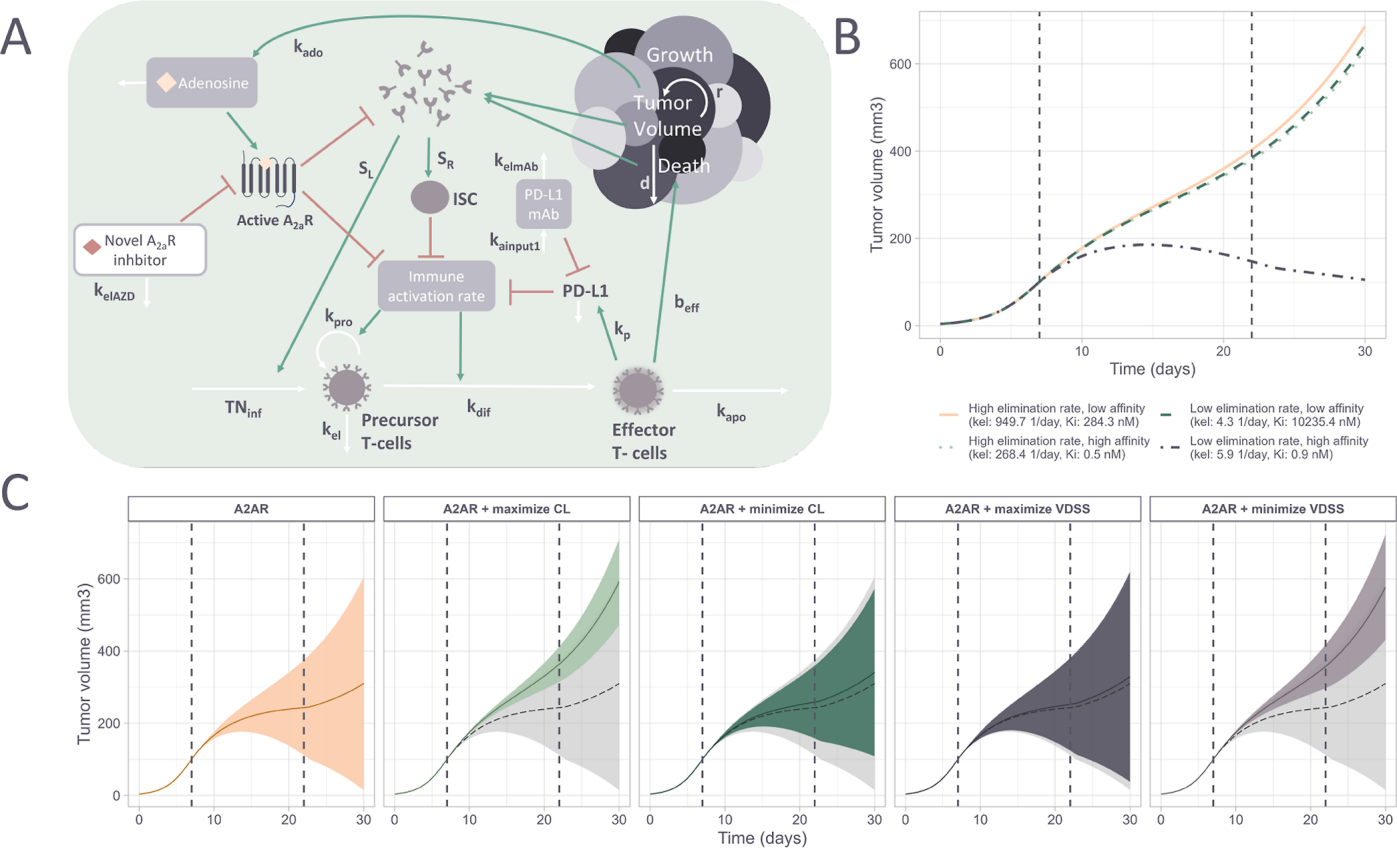
Simulated effect of *de novo* generated compounds on tumor volume in mice using a quantitative systems pharmacology (QSP) model. (A) A simplified graphical representation of the QSP model (figure and model adapted from Voronova et al.^18^), describing the relationship between the predicted concentration–time curve of the novel A_2A_R inhibitors and its inhibition of the immunosuppressive effect. White arrows indicate the dynamics of system components, such as the influx of precursor T-cells (TNinf) or tumor cell death (d). Red and green arrows indicate inhibition or stimulation of a model effect, respectively. (B) Simulations of four example generated compounds with extreme potency and elimination rate showing the tumor volume over time with the dosing interval between 8 and 23 days (dashed lines) (C) Line plots show the predicted tumor volume over time for all valid, unique, and within-applicability domain molecules from a set of 10,000 generated molecules. The colored shaded areas are the 90% prediction intervals, and the solid line is the mean prediction. The light gray shaded area and dashed lines represent the baseline scenario.

## Discussion

Finding compounds with both high potency and favorable PK is a key challenge in computational drug design, as well as the translation to PD. This work explored the direct inclusion of PK parameters as optimization criteria in a generative drug design framework. We compared *de novo* generated molecules with optimized potency for the A_2A_R to those with optimized potency and maximized or minimized CL, VDSS or FU. Subsequently, the scaled PK and affinity parameter estimates for the generated compounds were used in a previously published mice QSP model^18^ to compare the tumor inhibition efficacy of the predicted inhibitors. We found different PK optimization scenarios strongly influenced which molecules and scaffolds were preferentially generated. Moreover, the trends in the physicochemical properties of the generated molecules corresponded well with the relative difference between molecules in the PK data set. These results should be experimentally validated to confirm the utility of the PK integration in the generative framework prospectively.

One major limitation of this study was the limited predictive power of the QSPR models for human PK, which are known to be challenging to predict.^38–40^ The model for CL in particular showed weak predictivity (R^2^ values between 0.25 and 0.43 depending on the test set). One reason for this is that (human) *in vivo* PK data sets are relatively small and contain very diverse chemical structures compared to data sets for bioactivity prediction due to the difficulty and cost of data collection. Another reason is the complexity of the prediction tasks. Especially, CL is dependent on many different factors, such as the affinity for metabolizing enzymes, plasma protein binding and renal clearance. While it is difficult to compare to other published CL QSPR models directly due to varying data sets, and evaluation methods, *R* ^2^ values between 0.09 ^38^ (difficult, structurally dissimilar set) and 0.82 ^41^ have been reported, with most studies reporting values between 0.2 and 0.4.^38–40,42,43^ Lombardo et al.^38^ showed that molecules with primarily renal clearance could be better predicted. Therefore, it may be beneficial to discriminate between the different clearance mechanisms in future work. For example, through the use of a classification model that can predict the mechanism to filter the input for the CL prediction model. The FU model performs (R^2^ 0.51 and RMSE 0.21 (note. square root transformed)) better than the model for CL, but somewhat worse than another model described by Watanabe et al.^44^ (*R* ^2^ 0.72, RMSE 0.15) which was built on a larger data set (2738 compounds). Compared to another model published by Lombardo et al.^45^ (Geometric Mean Fold Error (GMFE) 1.87 on left-out structural-therapeutic classes), the VDSS model also performs worse (test set GMFE 2.17). The difference may be explained by the inclusion of predicted ionization state, which could be included in the future. The addition of multispecies or preclinical PK data may be beneficial to improve the predictivity of the model.^38,42,43,46^ The A_2A_R binding affinity model has a *R* ^2^ of 0.69 and RMSE of 0.64, which indicates satisfactory performance compared to the estimated noise in the public experimental data.^47,48^ This uncertainty is visualized by a scatterplot of individual values versus mean values for smiles where multiple measurements were available (Figure S1 with *R* ^2^ 0.89 and RMSE 0.43).

Another approach to improve the performance of the PK models could be through the use of physiologically based pharmacokinetic (PBPK) modeling. A recent comparison of noncompartmental analysis through the prediction of VDSS and CL was outperformed by a PBPK modeling approach, as well as direct prediction of the concentration–time curve and prediction of input parameters to a 2-compartment model.^49^ In addition to improving the generative modeling workflow, this may also enable more accurate simulations of the concentration–time profile. A further advantage of PBPK models would be the possibility to use *in vitro* end points such as intrinsic clearance for the QSPR models, which would facilitate the experimental validation of integration of the PK in DrugEx.

A well-defined applicability domain is essential for the reliable application of QSAR/QSPR models.^50^ This holds especially in this context, where multiple QSPR models were built on different data sets, which thus have different applicability domains. Here, the applicability domain was defined based on an implementation of the TOPKAT OPS.^28,29^ This method was shown to discriminate well between inliers and outliers for the current tasks through bootstrapping analysis where higher/lower performance was observed for the inliers and outliers compared to the complete test sets respectively. However, a disadvantage of including the applicability domain as an objective in reinforcement learning is that the ability of DrugEx to explore new chemical space is restricted. There are many different definitions and methods to determine the applicability domain of a QSPR model, using a different applicability domain may increase the coverage of the generative model.^51^ Another option could be to use proteochemometric or multitask models to leverage data from other proteins/tasks.^52^

Even though a considerable fraction (>*0.4*) of the generated molecules is within the applicability domain of all relevant QSPR models, this fraction does not increase during reinforcement learning in every scenario. This indicates that the generator model does not learn how to generate molecules within the applicability domain well. A reason for this might be the binary nature of the “applicability domain tasks”, therefore, implementing the applicability domain as a continuous objective might be beneficial in the context of reinforcement learning. This could, for example, be achieved by using the Mahalanobis distance of the TOPKAT OPS directly, rather than an empirical cutoff, to improve feedback to DrugEx.

Quantification of the uncertainty in the QSPR model predictions will be important in the broader relevance of the framework. While performance metrics on the test set and the bootstrapping analysis can estimate the average prediction error, quantifying the uncertainty on individual predictions would be highly desirable. This may be achieved through conformal prediction.^38,53,54^ Another advantage of conformal prediction in this generative drug design framework could be to create an active learning loop, by identifying compounds to synthesize and test to further expand the applicability domain of the QSPR models. While it is out of the scope of the current work, it should be noted that the field will profit significantly from an increase in experimental validation. It is well-known that the mean error of high-quality public data (*K*_*i*_ values) is 0.44 p*K*(*i*) units and slightly larger for IC50 values.^47^ An increase in experimental validation, with a focus on publication of activity values of molecules that were selected with computational methods to be desirable and showed low to little affinity, will increase predictive model quality and uncertainty estimation.

The drug efficacy of the generated compounds was evaluated through a QSP model. Notably, the highest mean tumor growth inhibition on day 30 was for the molecules generated in the maximize potency-only scenario. This indicates that a mean loss of potency is not compensated by higher drug exposure (lower clearance or higher volume of distribution) for this case study. However, it is difficult to draw quantitative conclusions from the current framework. First, the QSP model predictions add additional uncertainty on top of the QSPR parameter predictions. Second, the allometric scaling also adds an uncertain amount of error to these predictions. This further confirms the need of classifying the uncertainty of each prediction.

In this work, we simulated the effect of either maximization or minimization of a PK property through reinforcement learning with a QSP model with a fixed dosing regimen to evaluate how *in vivo* efficacy of generated molecules in different scenarios compared. However, the results may have changed if we allowed the dosing to be dynamic with constraints on the amount and frequency to correct for drug exposure. A clinically relevant question could be what the minimum required dose of a drug would be to achieve a predefined efficacy level. Then, the objectives in reinforcement learning could be to both optimize the drug potency and drug exposure. Another option would be to build on the pipeline proposed by Chen et al.^3^ where (among other applications) an optimal molecular space is defined through the determination of the PK driver for efficacy using virtual enumeration of a QSP model. Here the optimal molecular space could be used to determine the optimization criteria for DrugEx, in essence reverting the workflow as described in this paper.

The main focus of this paper was to investigate the effect of combined reinforcement learning for pharmacokinetics and potency and the trade-off between them. However, there are other important criteria in hit identification, including the synthesizability of a drug, which were not taken into account here. Some generated molecules displayed unstable or reactive properties that would be infeasible to synthesize. For future applications of this pipeline, it is important to take synthesizability into account as well through measures such as the Synthetic Accessibility score,^55^ Retrosynthetic Accessibility score^56^ or LED3 score.^57^ Finally, while this paper focuses on the A_2A_R, the described workflow could be applied to any other target of interest for which there is sufficient bioactivity data available. The quality of the generated compounds depends on the quantity, diversity, and quality of the data used to train the QSAR models.

## Conclusion

PK and PD are inherently linked and are therefore both important optimization criteria in drug discovery. In this proof-of-concept, we have demonstrated how we can capture trends in PK characteristics while simultaneously optimizing the potency of generated compounds through the reinforcement-learning generative drug design framework DrugEx. In addition, we have shown how the effect of different PK and potency optimization criteria may be understood through QSP modeling. For practical exploitation of this framework, the results should be experimentally validated. An important limitation of this work is the limited performance of the QSPR model for CL. In future work, we will refine this pipeline through uncertainty quantification and improve the efficacy simulations through PBPK modeling.

## Supplementary Material



## Data Availability

All data for the analysis presented in this manuscript is available on Zenodo (https://zenodo.org/records/15082627). The code is available at https://github.com/CDDLeiden/PK-in-generative-drug-design.

## Notes

Additional figures and tables of data distribution, hyperparameter grid search results, QSPR model feature importance, finetuning and reinforcement learning optimization, results for reinforcement learning with optimization for PK and QSP model equations and parameters (PDF)
