# Supplementary material for: Integrating Pharmacokinetics and Quantitative Systems Pharmacology Approaches in Generative Drug Design
Source: J Chem Inf Model. 2025 May 9;65(10):4783–96. doi: 10.1021/acs.jcim.5c00107 (PMC12117666; doi:10.1021/acs.jcim.5c00107)
Supplement: Supplementary file 1 [file ci5c00107_si_001.pdf]

Supporting Information: Integrating Pharmacokinetics and Quantitative Systems Pharmacology Approaches in Generative Drug Design

Helle W. van den Maagdenberg,[†] Jikke de Mol van Otterloo,[†] J.G. Coen van Hasselt,[†] Piet H. van der Graaf,[†,‡] and Gerard J.P. van Westen[*,†]

†*Leiden Academic Centre of Drug Research, Leiden University, 2333 CC Leiden, The Netherlands*

‡*Certara, CT2 7FG, Canterbury, UK.*

E-mail: gerard@lacdr.leidenuniv.nl

Table S1: Threshold for clipped score modifier based on the 10th and 90th percentiles of the property values in the respective datasets.

**Objective**	**Minimum Threshold**	**Maximum Threshold**	**Unit**
Maximize $A_{2A}R$ pKi	5.5	8.6	-log(M)
Minimize FU	0.95	0.10	sqrt fraction
Maximize FU	0.10	0.95	
Minimize VDSS	0.95	-0.74	log(L/kg)
Maximize VDSS	-0.74	0.95	
Minimize Cl	1.34	-0.13	log(mL/min/kg)
Maximize Cl	-0.13	1.34	

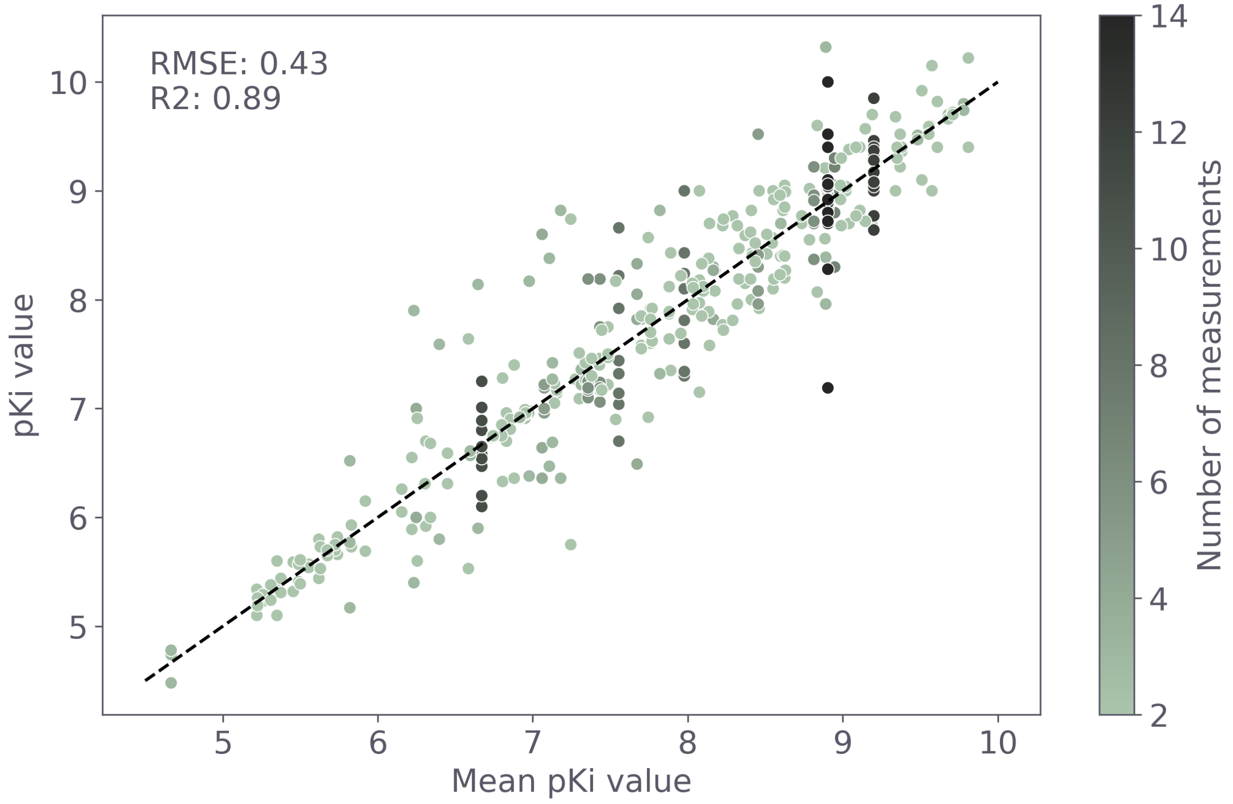

Figure S1: Variance of experimental $A_{2A}R$ pKi measurements in the Papyrus $A_{2A}R$ datasets. The mean value of any compound with more than one pKi value in the dataset is plotted against the individual measurement. Data points with identical pKi (potential duplicates) were not included. The points are colored by the number of measurements for each compound.

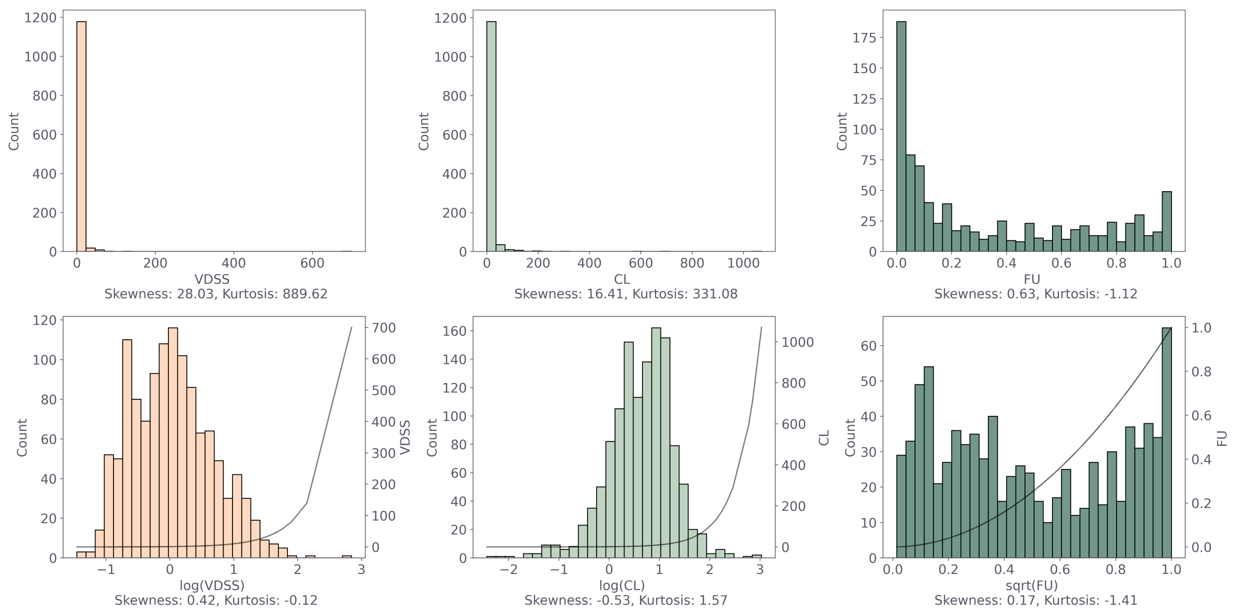

Figure S2: Distribution of the non-transformed (first row) and transformed (second row) volume of distribution at steady state (left), clearance (middle) and unbound fraction (right) data.

Table S2: Results of hyper parameter grid search

Target	Algorithm	Features	Filters	Hyperparameters	mean R2 (sd)
	**KNeighborsRegressor**	MorganFP, RDkit	high_corr_th: 0.99, boruta_perc: 80	n_neighbors: 5	0.64 (0.02)
**$A_{2A}R$**	**PLSRegression**	MorganFP	high_corr_th: 0.90, boruta_perc: 80	n_components: 2	0.53 (0.02)
	**RandomForestRegressor**	MorganFP	high_corr_th: 0.90, boruta_perc: 80	max_depth: 20, n_estimators: 100	0.66 (0.01)
	**SVR***	MorganFP, RDkit	high_corr_th: 0.99, boruta_perc: 80	C: 10, kernel: rbf	0.69 (0.02)
	**ChemProp**			depth: 3, dropout: 0.1, ffn_num_layers: 2, hidden_size: 512	0.63 (0.03)
	**KNeighborsRegressor**	MorganFP, RDkit	high_corr_th: 0.95, boruta_perc: 100	n_neighbors: 10	0.51 (0.08)
**FU**	**PLSRegression**	MorganFP, RDkit	high_corr_th: 0.90, boruta_perc: 80	n_components: 2	0.57 (0.05)
	**RandomForestRegressor**	MorganFP, RDkit	high_corr_th: 0.95, boruta_perc: 80	max_depth: 20, n_estimators: 1000	0.60 (0.06)
	**SVR**	MorganFP, RDkit	high_corr_th: 0.99, boruta_perc: 80	C: 1, kernel: rbf	0.65 (0.04)
	**ChemProp**			depth: 5, dropout: 0.0, ffn_num_layers: 2, hidden_size: 256	0.56 (0.05)

VDSS	**KNeighborsRegressor**	MorganFP, RDkit	high_corr_th: 0.99, boruta_perc: 100	n_neighbors: 5	0.44 (0.02)
	**PLSRegression**	MorganFP, RDkit	high_corr_th: 0.90, boruta_perc: 80	n_components: 2	0.47 (0.05)
	**RandomForestRegressor**	MorganFP, RDkit	high_corr_th: 0.90, boruta_perc: 80	max_depth: 20, n_estimators: 1000	0.51 (0.03)
	**SVR**	MorganFP, RDkit	high_corr_th: 0.99, boruta_perc: 80	C: 1, kernel: rbf	0.51 (0.04)
	**ChemProp**			depth: 3, dropout: 0.2, ffn_num_layers: 3, hidden_size: 128	0.49 (0.05)
CL	**KNeighborsRegressor**	MorganFP, RDkit	high_corr_th: 0.90, boruta_perc: 80	n_neighbors: 7	0.25 (0.06)
	**PLSRegression**	MorganFP	high_corr_th: 0.90, boruta_perc: 80	n_components: 2	0.29 (0.04)
	**RandomForestRegressor**	MorganFP	high_corr_th: 0.90, boruta_perc: 80	max_depth: 20, n_estimators: 1000	0.28 (0.04)
	**SVR**	MorganFP, RDkit	high_corr_th: 0.95, boruta_perc: 80	C: 1, kernel: rbf	0.31 (0.05)
	**ChemProp**			depth: 3, dropout: 0.2, ffn_num_layers: 3, hidden_size: 128	0.16 (0.05)

*Grid search for SVR models for $A_{2A}R$ with low_var_th: 0.01 did not complete within the set 8-hour maximum run time.

Table S3: Result of grid search of epsilon and mutation network hyperparameters for DrugEx RNN reinforcement learning Best model (bold) is determined on geometric mean (GM) of modified predicted $A_{2A}R$ and FU scores for all valid, unique, novel (not in PK or $A_{2A}R$ dataset) and applicable molecules over the total number of generated compound. The arithmetic means of separate scores are also provided as well as the average minimum tanimoto (MorganFP radius 3, bits 2048) distance to other molecules.

**Epsilon**	**Mutate model**	**Validity**	**Uniqueness**	**Novelty**	**GM scores**	**A2AR score**	**FU score**	**A2AR AP**	**FU AP**	**Minimum distance**
0.01	finetuned	1.00	0.21	0.21	0.07	0.17	0.18	0.09	0.20	0.20
0.01	pretrained	1.00	0.18	0.18	0.08	0.15	0.16	0.09	0.17	0.23
0.05	finetuned	0.99	0.50	0.50	0.16	0.36	0.42	0.22	0.48	0.28
0.05	pretrained	0.99	0.73	0.71	0.23	0.47	0.56	0.33	0.69	0.34
**0.1**	**finetuned**	**0.98**	**0.69**	**0.67**	**0.25**	**0.36**	**0.53**	**0.39**	**0.65**	**0.39**
0.1	pretrained	0.97	0.88	0.85	0.23	0.41	0.60	0.40	0.81	0.51
0.2	finetuned	0.97	0.80	0.76	0.22	0.30	0.56	0.43	0.72	0.48
0.2	pretrained	0.95	0.97	0.93	0.09	0.26	0.52	0.28	0.83	0.63
0.3	finetuned	0.96	0.83	0.76	0.18	0.26	0.49	0.41	0.72	0.53
0.3	pretrained	0.95	0.99	0.94	0.06	0.23	0.45	0.23	0.81	0.66

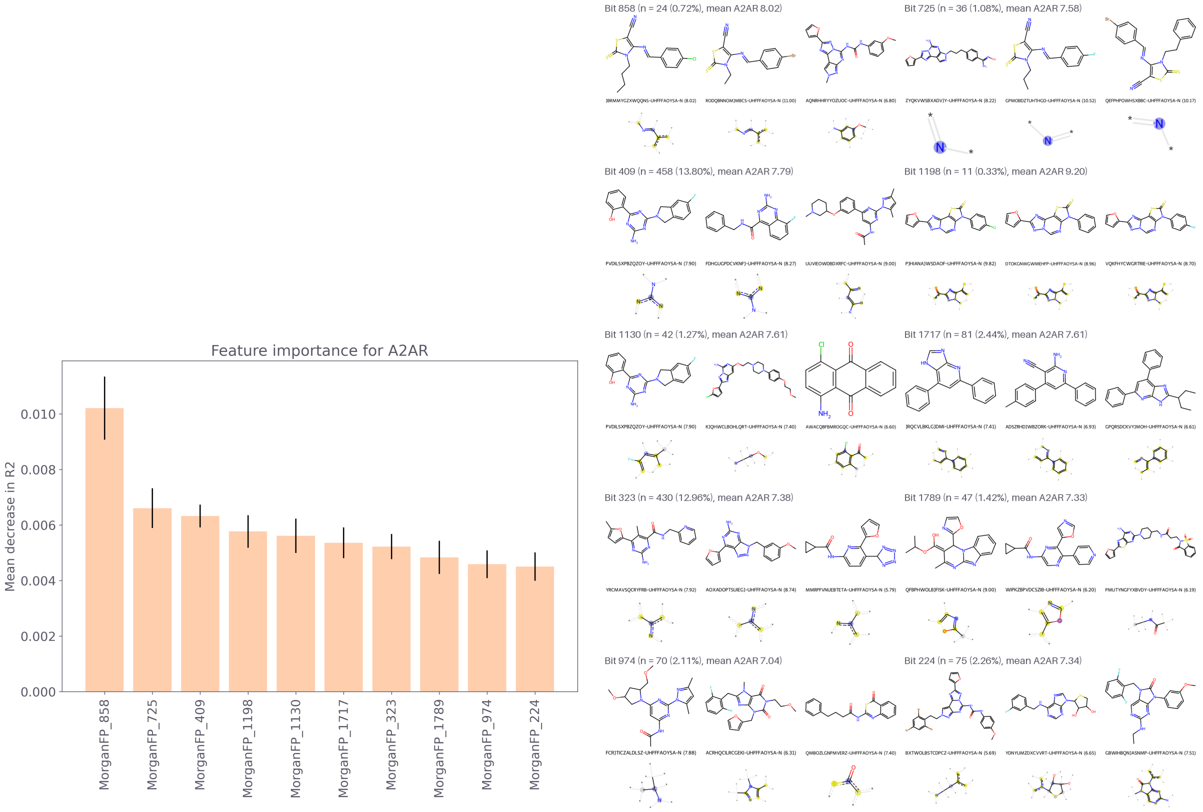

(a) Feature importance for the A2AR QSPR model

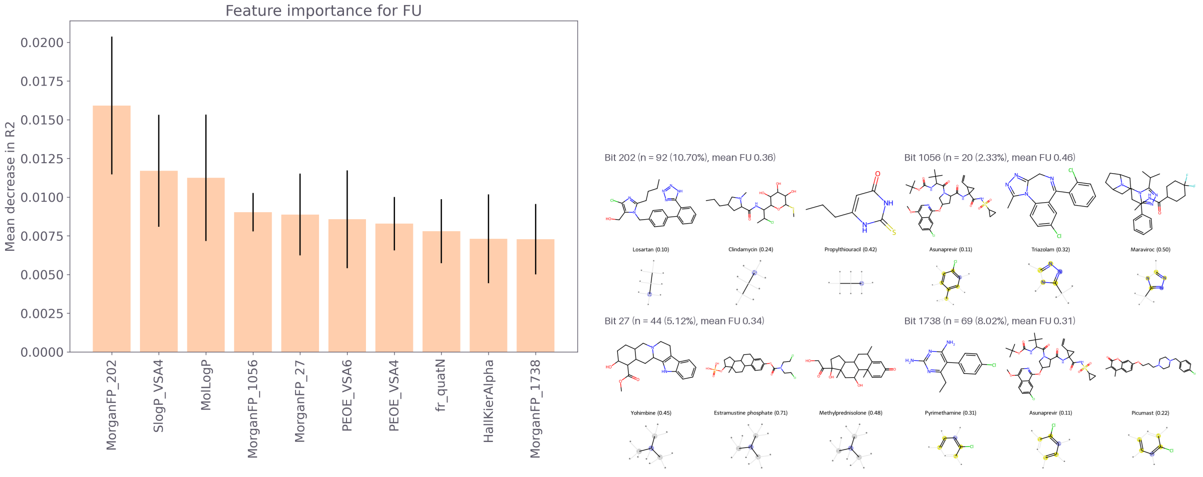

(b) Feature importance for the FU QSPR model

Figure S3: Permutation feature importance of 10 most important features for each QSPR model. Every sub-figure shows a bar plot with the average reduction in R^2 and error bars representing the standard deviation. For Morgan fingerprint bits (radius 3, bits 2048) in the most important feature list, three molecules were randomly selected from the dataset to visualize the bits. The bit figures also show occurrence and the average respective property values for molecules containing the bit. As well as the property value of the displayed molecules.

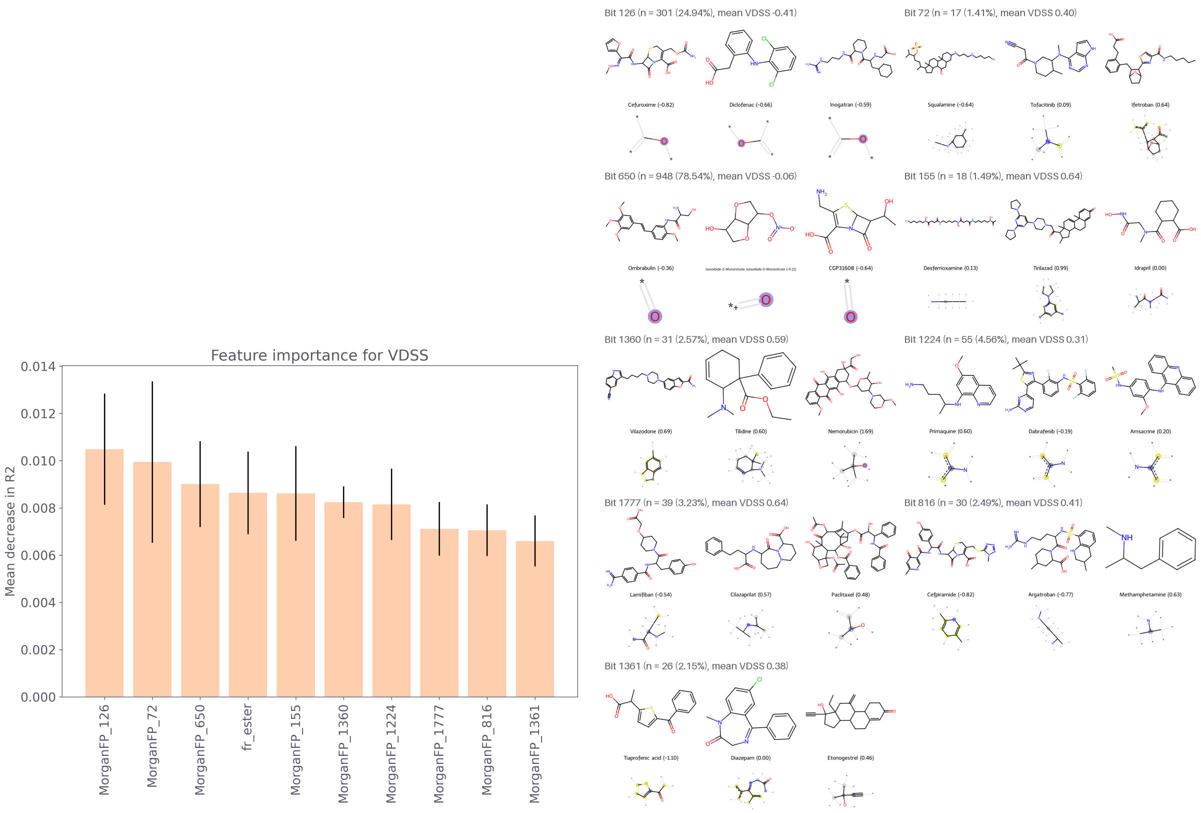

(c) Feature importance for the VDSS QSPR model

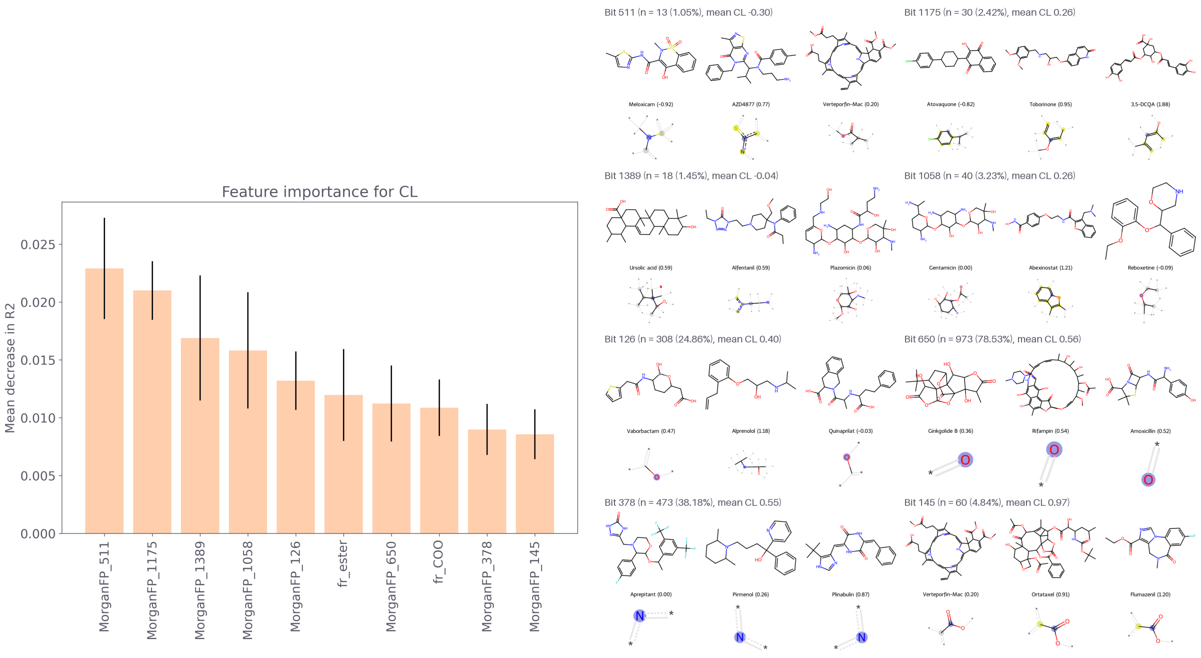

(d) Feature importance for the CL QSPR model

Figure S3: (Continued) Permutation feature importance of 10 most important features for each QSPR model.

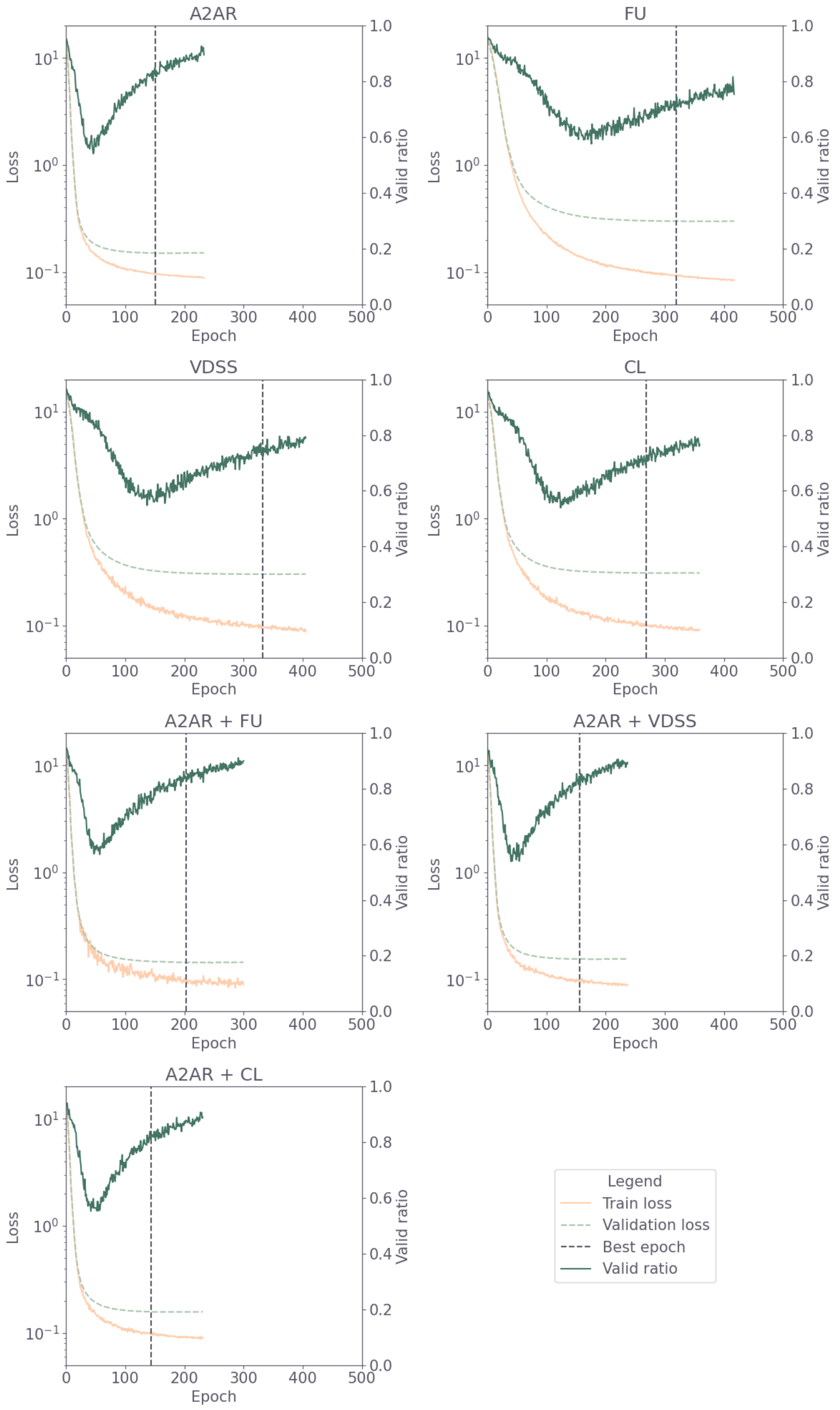

Figure S4: Finetuning optimization trajectories of DrugEx RNN models on different datasets. Facet titles indicate which dataset was trained on. For individual objectives, the model was trained on the whole respective dataset. For combinations of the $A_{2A}R$ dataset with a PK dataset, the model was finetuned on all compounds in the $A_{2A}R$ dataset that are applicable according to the optimal prediction space for both datasets.

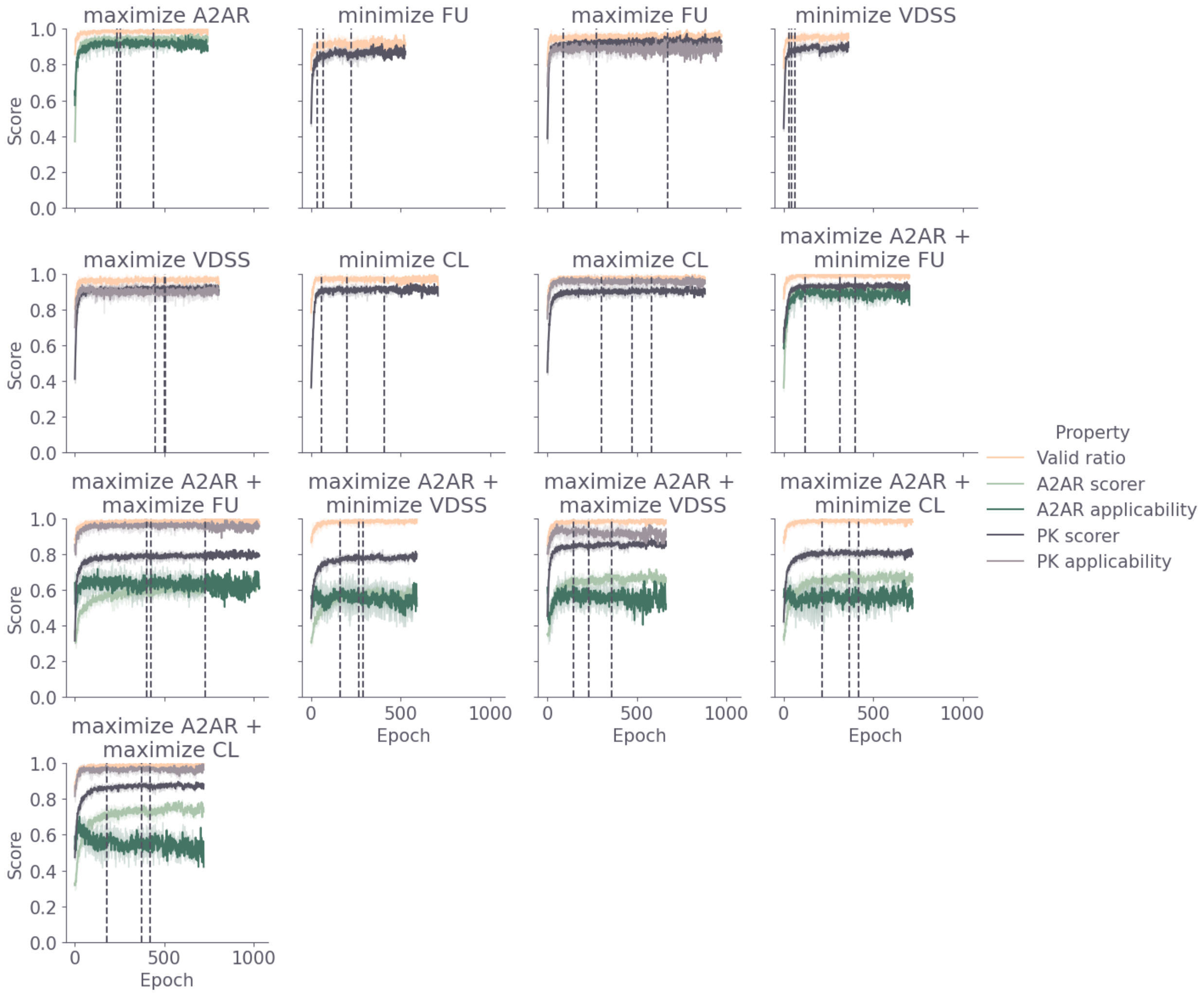

Figure S5: Reinforcement learning optimization trajectories of DrugEx RNN models with varying sets of optimization criteria. Each facet shows the average scores (y-axis) of 200 generated molecules per epoch (x-axis) for the mean of the three replicates. The shaded area shows the 95% confidence interval. Vertical dashed lines indicate the best epoch for each of the three replicates. Facet titles list the optimization criteria.

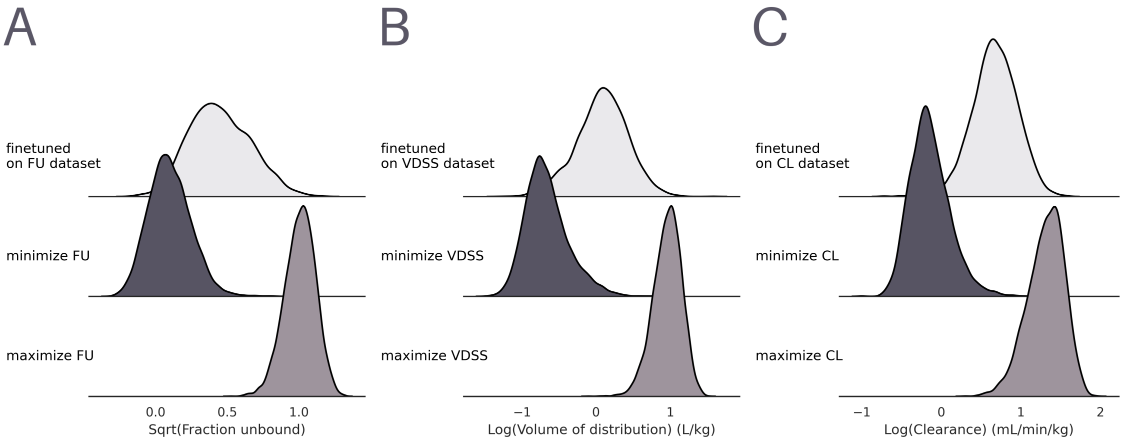

Figure S6: Distribution of scores of all valid and unique molecules from a set of 10000 generated molecules for different DrugEx optimization scenarios. The panels show optimization for minimization/maximization of (A) FU, (B) VDSS, and (C) CL.

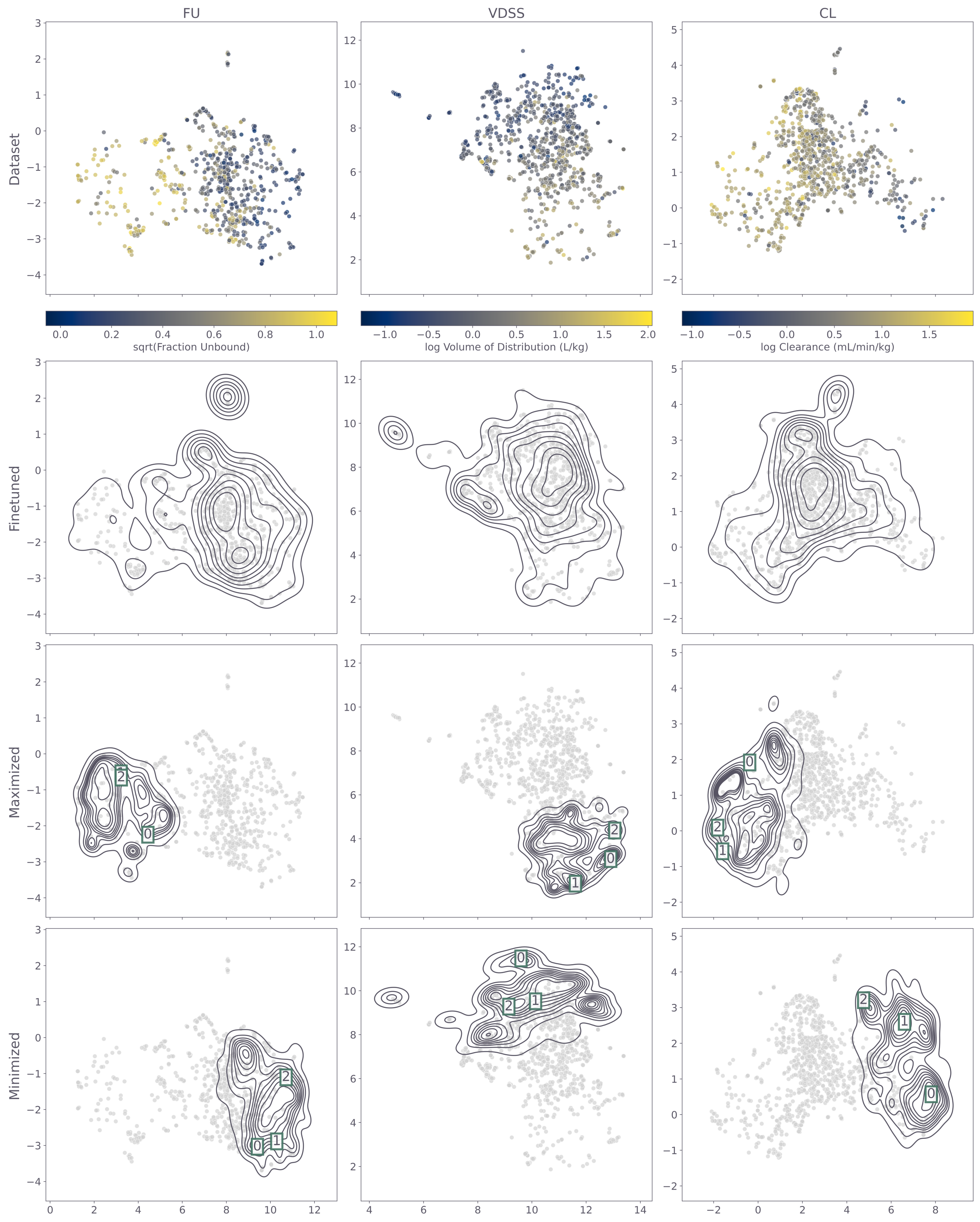

Figure S7: Umap representation of the pharmacokinetics dataset overlapping with the density of the generated molecules from the trained DrugEx generators with different objectives. The columns represent the different properties. The first row shows the dataset colored by the respective properties from left to right: square root FU, log VDSS, log CL; containing only the molecules applicable for that objective. The second row shows the density of all valid and unique molecules generated by the fine-tuned models. The third and fourth row show the density of the unique and valid generated molecules maximization or minimization of a pharmacokinetics property, respectively.

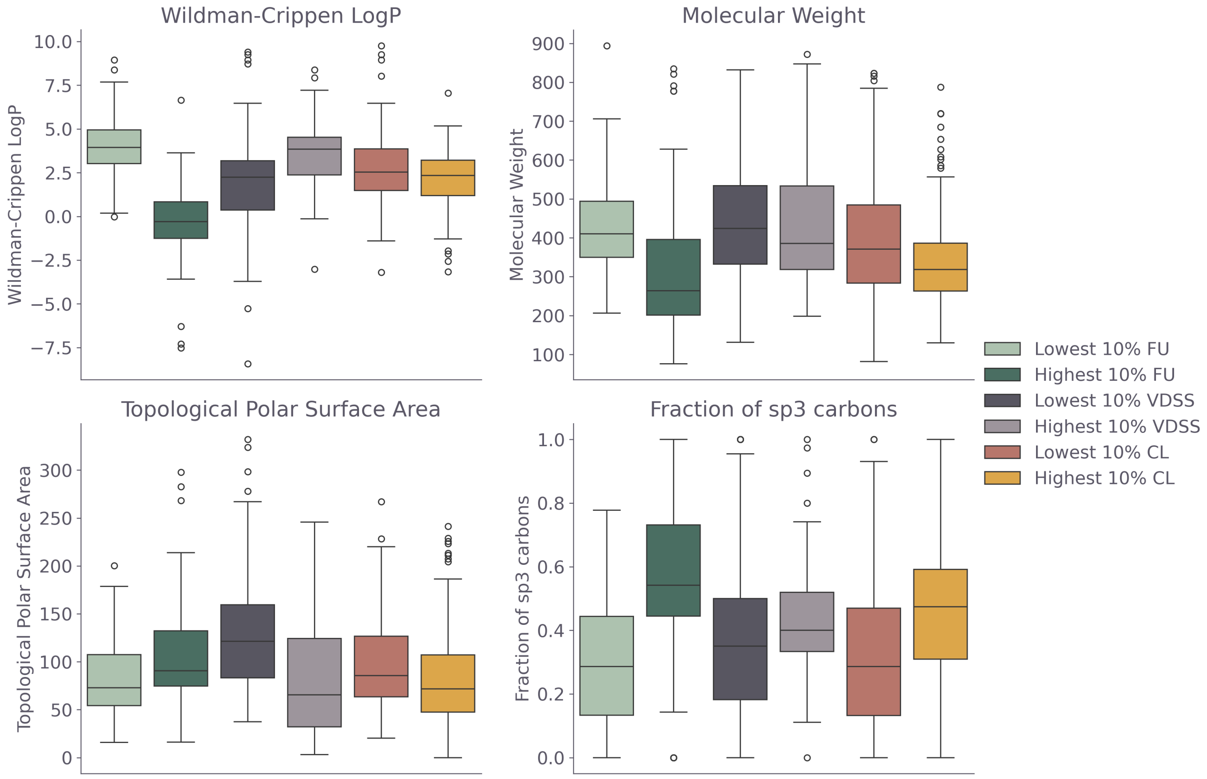

Figure S8: Box plots of the physicochemical properties (Wildman-Crippen LogP, Molecular Weight, Topological Polar Surface Area, Fraction of sp^3-hybridized carbons) of bottom 10 percentile and top 10 percentile PK dataset.

Figure S9: QSP model equations, adapted from Voronova et al.[S1] Variable and parameter definitions are listed in table S4 and S5, respectively.

$$\frac{d}{dt}(Ad1) = -kainput1 * Ad1 \tag{1a}$$

$$\frac{d}{dt}(Ac1) = kainput1 * Ad1 * convF1 - kelmab * Ac1 \tag{1b}$$

$$Cc1 = \frac{Ac1}{Vc} \tag{1c}$$

$$\frac{d}{dt}(Ac2) = -kel_{ARinh} * Ac2 \tag{1d}$$

$$Cc2 = \frac{Ac2}{MW * Vc} * 10^6 \tag{1e}$$

$$PDL1free = PDL1/(1 + \frac{Cc1}{Kd1}) \tag{1f}$$

$$A2ARoccup = \frac{Ado * sCf}{Kd_{ado}}/(1 + \frac{Ado * sCf}{Kd_{ado}} + \frac{Cc2}{Kd_{ARinh}}) \tag{1g}$$

$$Ado_{suppr} = Vmax_{supr} * \frac{A2ARoccup}{A2ARoccup + IC50} \tag{1h}$$

$$TKR = (beff * CTL + d) \tag{1i}$$

$$Ag = TKR * Tum * (1 - Ado_{suppr}) \tag{1j}$$

$$PRfunc = (1 - PDL1free) * (1 - \frac{Ag}{Ag + sR}) * (1 - Ado_{suppr}) \tag{1k}$$

$$TNinf = kLn * \frac{Ag}{Ag + sL} \tag{1l}$$

$$CD8_{tot} = CTL + TN \tag{1m}$$

$$ISC = \frac{Ag}{Ag + sR} \tag{1n}$$

$$Tum_{gr} = Tum * r * (1 - \frac{Tum}{TVmax}) * xf \tag{1o}$$

$$Tum_{kill} = (beff * CTL + d) * Tum \tag{1p}$$

$$CTL_{dynamic1} = TNinf + kpro * PRfunc * TN - kel * TN \tag{1q}$$

$$CTL_{dynamic2} = kdif * PRfunc * TN \tag{1r}$$

$$CTL_{dynamic5} = kapo * CTL \tag{1s}$$

$$\frac{d}{dt}(Tum) = Tum_{gr} - Tum_{kill} \tag{1t}$$

$$\frac{d}{dt}(TN) = CTL_{dynamic1} - CTL_{dynamic2} \tag{1u}$$

$$\frac{d}{dt}(CTL) = CTL_{dynamic2} - CTL_{dynamic5} \tag{1v}$$

$$\frac{d}{dt}(PDL1) = \frac{CTL}{CTL + Kp} - PDL1 \tag{1w}$$

$$\frac{d}{dt}(Ado) = Vado * \frac{Tum}{Tum + Kado} - Ado \tag{1x}$$

$$\tag{1y}$$

Table S4: QSP model variable names, units and descriptions, adapted from Voronova et al.[S1]

Variable	Unit	Description
Ad1	mg	mAb administration cmt
Ac1	nmol	mAb central cmt
Ac2	mg	$A_{2A}R$ inhibitor central cmt
Cc1	nM	mAb
Cc2	nM	$A_{2A}R$ inhibitor free
PDL1free		Fraction unbound PDL1
A2ARoccup		Ado occupied fraction of $A_{2A}R$ receptors
Ado_{suppr}		Effect of ado-dependent $A_{2A}R$ occupancy on the activity of dTeff and APC
TKR	1/d	Tumor shrinkage rate
Ag	uL/d	Systemic antigen levels
PRfunc		Proliferation Rate/Immune activation rate
TNinf	cells/day	CD8 precursor influx to tumor
$CD8_{tot}$	cells	Total CD8+ T cells
ISC		Immunosuppressive cells
Tum_{gr}	uL/d	Tumor growth rate
Tum_{kill3}	uL/d	Tumor cell death
$CTL_{dynamic1}$	cells/d	Influx of naive T cells
$CTL_{dynamic2}$	cells/d	Differentiation of naive T cells
$CTL_{dynamic5}$	cells/d	Apoptosis of CTL
Tum	uL	Tumor volume
TN	cells	Naive T cells
CTL	cells	Cytotoxic T-lymphocytes
PDL1	nM	PDL1
Ado	nM	Adenosine concentration

Table S5: List of the QSP model parameters, adapted from Voronova et al.[S1] Parameter names are given as in provided model code.

Parameter	value	unit	Description
TVmax	3500	uL	Maximal size of tumor
beff	0.001	1/(d*cell)	Rate of tumor cell kill by dTeff
r	0.522	1/d	Tumor growth rate
kLn	209.674	cells/d	Maximal influx rate of nTeff cells
$sL * exp(\beta_{CIV151})$	$4.556 * exp(0)$	uL/d	T cell infiltration tumor under Ag exposure
Kp	1279.917	cells	Sensitivity of PD-L1 expression up-regulation to dTeff count
Kado	80	1/d	Adenosine accumulation rate constant
IC50	1.823		The $A_{2A}R$ occupancy with a 50% decrease in immune cell activity
$sR * exp(\beta_{MCA205})$	$57.052 * exp(0.531)$	uL/d	Sensitivity of cellular immunosuppression to build-up of systemic Ag
sCf	1		Scaling factor of total intratumoral adenosine to extracellular adenosine levels
$Vado * exp(\beta_{MCA205})$	$100000 * exp(-3)$	nM	Adenosine level in tumor
$TVin * exp(\beta_{MCA205})$	$2.055 * exp(0.69)$	uL	Initial tumor volume
Vc_{ARinh}	-	L	Central compartment VDSS of a simulated $A_{2A}R$ inhibitor
Kd_{ARinh}	-	nM	Binding affinity of a simulated $A_{2A}R$ inhibitor
kel_{ARinh}	-	1/d	Elimination rate of a simulated $A_{2A}R$ inhibitor
MW	-	g/mol	Molecular weight of a simulated $A_{2A}R$ inhibitor
convF1	6.66		Conversion factor for mAb in mg to nmol
kainput1	8	1/d	mAb i.p.absorption rate
Vc	0.003	L	mAb volume of distribution
kelmAb	0.1	1/d	mAb elimination
Kd_{ado}	1182	nM	Affinity of ado for A2AR
Kd1	30	nM	mAb/PD-L1 binding affinity
kel	0.2	1/d	Half-life of Tn naive ~10 days
kapo	2.0	1/d	Half-life of CTL ~1 days
kpro	3	1/d	Maximal T cells proliferation rate
kdif	3.2	1/d	Maximal T cells differentiation rate
d	0.01	1/d	Slow "spontaneous" tumor cells death rate
$Vmax_{supr}$	0.7		The maximal adenosine effect on dTeff and APC suppression
xf	-		Stops tumor growth if tumor volume smaller than 10 and time at least 7 days

References

(S1) Voronova, V.; Peskov, K.; Kosinsky, Y.; Helmlinger, G.; Chu, L.; Borodovsky, A.; Woessner, R.; Sachsenmeier, K.; Shao, W.; Kumar, R.; Pouliot, G.; Merchant, M.; Kimko, H.; Mugundu, G. Evaluation of Combination Strategies for the A2AR Inhibitor AZD4635 Across Tumor Microenvironment Conditions via a Systems Pharmacology Model. *Frontiers in Immunology* **2021**, *12*, 617316.
